# Fire Intumescent, High-Temperature Resistant, Mechanically Flexible Graphene Oxide Network for Exceptional Fire Shielding and Ultra-Fast Fire Warning

**DOI:** 10.1007/s40820-022-00837-1

**Published:** 2022-04-06

**Authors:** Cheng-Fei Cao, Bin Yu, Zuan-Yu Chen, Yong-Xiang Qu, Yu-Tong Li, Yong-Qian Shi, Zhe-Wen Ma, Feng-Na Sun, Qing-Hua Pan, Long-Cheng Tang, Pingan Song, Hao Wang

**Affiliations:** 1 Centre for Future Materials, University of Southern Queensland, Springfield Central, 4300 Australia; 2 State Key Laboratory of Fire Science, University of Science and Technology of China, Hefei, 230026 China; 3 College of Material, Chemistry and Chemical Engineering, Key Laboratory of Organosilicon Chemistry and Material Technology of MoE, Hangzhou Normal University, Hangzhou, 311121 China; 4 College of Environment and Resources, Fuzhou University, Fuzhou, 350116 China; 5 School of Engineering, Zhejiang A & F University, Hangzhou, 311300 China

**Keywords:** Graphene oxide, Multi-amino molecule, Flame resistance, Fire intumescent effect, Ultra-fast fire warning

## Abstract

Smart fire alarm sensor (FAS) materials with mechanically robust, excellent flame retardancy as well as ultra-sensitive temperature-responsive capability are highly attractive platforms for fire safety application. However, most reported FAS materials can hardly provide sensitive, continuous and reliable alarm signal output due to their undesirable temperature-responsive, flame-resistant and mechanical performances. To overcome these hurdles, herein, we utilize the multi-amino molecule, named HCPA, that can serve as triple-roles including cross-linker, fire retardant and reducing agent for decorating graphene oxide (GO) sheets and obtaining the GO/HCPA hybrid networks. Benefiting from the formation of multi-interactions in hybrid network, the optimized GO/HCPA network exhibits significant increment in mechanical strength, e.g., tensile strength and toughness increase of ~ 2.3 and ~ 5.7 times, respectively, compared to the control one. More importantly, based on P and N doping and promoting thermal reduction effect on GO network, the excellent flame retardancy (withstanding ~ 1200 °C flame attack), ultra-fast fire alarm response time (~ 0.6 s) and ultra-long alarming period (> 600 s) are obtained, representing the best comprehensive performance of GO-based FAS counterparts. Furthermore, based on GO/HCPA network, the fireproof coating is constructed and applied in polymer foam and exhibited exceptional fire shielding performance. This work provides a new idea for designing and fabricating desirable FAS materials and fireproof coatings.

**Supplementary Information:**

The online version contains supplementary material available at 10.1007/s40820-022-00837-1.

## Highlights

- Graphene oxide-based hybrid networks were fabricated via introducing multi-amino molecule with triple roles (i.e., cross-linker, fire retardant and reducing agent).
- The optimized hybrid network with mechanically robust, exceptional intumescent effect and ultra-sensitive fire alarm response (~ 0.6 s) can be used as desirable smart fire alarm sensor materials.
- Exceptional fire shielding performances, e.g., ~ 60% reduction in peak heat release rate and limiting oxygen index of ~ 36.5%, are achieved, when coated such hybrid network onto combustible polymer foam.

**Supplementary Information:**

The online version contains supplementary material available at 10.1007/s40820-022-00837-1.

## Graphical abstract

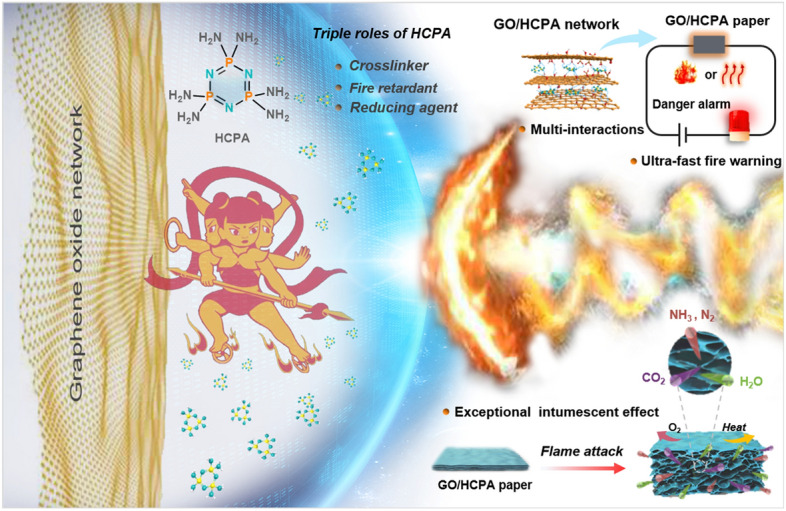

## Introduction

As we all know, utilization of fire is one of significant symbols that our ancestors from barbarism into human's civilization. It is indisputable that the development and progress of human society are accompanied by the control and utilization of fire [1]. However, fire is a double-edged sword, once it is out of control, it may bring potential fire hazards and further cause serious fire accidents, and poses great threatens to the human life, society and ecological environment [2]. Especially, in recent few decades, with the rapid development of industrialization, numerous varieties of polymer materials were synthesized and produced and have been extensively applied in many fields [3–7]. Since most polymeric materials have inherent flammability, upon ignited, they burn rapidly and lead to serious fire disasters. Thus, to some extent, extensive application of combustible polymer materials increases the fire accident frequency [8].

Among polymeric materials, polymer foam materials such as polyurethane (PU) foams have been widely used for building energy-saving [9, 10]. Unfortunately, large specific surface area and high organic components render its highly flammable, which poses potential fire safety hazards. In fact, there are several fire accidents that induced by inflammable PU foam materials every year around the world [11]. Among the above fire disasters caused by combustible polymer foam materials, the London’s Grenfell Tower fire, which occurred on June 14, 2017, could be most representative. It is reported that this tragic accident led to 71 people deaths and the main reason is that the exterior cladding of this building is made of flammable polymer foam materials instead of a fireproof alternative [9, 12]. Besides, the fast spread of flame in fire accident is another key factor resulting heavy casualties. The results show that once fire accident occurred, the flame can spread to almost whole building within 10 min, as a result, there was not enough time for those people in building to tackle fire or escape, thus leading to this fire disaster [13, 14]. Therefore, it is a considerable global challenge for human to reduce or avoid the occurrence of fire accidents induced by combustible polymers.

Flame-retardant technology is a key effective strategy to reduce the fire risk, and it includes a series of flame-retardant methods, e.g., modification of polymer, addition of flame-retardant fillers and surface coating, which is aiming to improve various combustible materials. In addition, considering that the highly flammability of polymer foam materials and flame spread speed in real fire accidents, fire alarm systems also play an important role in fire safety and prevention. Apart from existing conventional fire alarm detectors, e.g., smoke, temperature, and infrared detectors, recently, a series of fire alarm sensors (FASs) have been designed and achieved based on various working mechanisms and sensor materials. For instance, Tang’s group firstly reported a smart silicon resin/graphene oxide coating on combustible PU substrate [15]. Such coating can provide excellent flame retardancy and protect graphene oxide (GO) sheets from thermal decomposition during combustion process. More importantly, based on thermal reduction mechanism of GO sheets, fire alarm signal can be triggered within 2–3 s once encountered flame attack. Later, other FAS systems have been gradually designed and developed, such as color-changing molecular sensor [16], phase-changing/shape-changing sensors [17, 18] and thermoelectric sensors [19–21]. Therefore, a variety of fire sensor materials are designed, fabricated and employed, e.g., carbon nanotube, graphene derivatives, metal oxide, MXene, nanocarbon black, chitosan and other biomass-based materials [22–30]. Overall, both above FAS show satisfying fire warning performance, e.g., for most reported works, a desirable flame response time of < 5 s can be obtained, which is superior to existing commercial fire alarm devices, e.g., smoke and infrared detectors with a > 100 s fire response time [31, 32]. Among above FAS systems, GO-based FASs are most attractive and have been widely studied and reported. On the one hand, by combining with other flame retardants, it can be used as effective flame-retardant coating. On the other hand, sensitive fire warning performance can obtain based on rapid thermal reduction of GO network under flame or high-temperature conditions [33]. More importantly, abundant oxygen-containing groups on GO sheets provide the possibility of chemical modification and render it possesses good designability, e.g., it can exist in the form of various shapes (including coating, paper or film and aerogel [34–37], etc.). Usually, pure GO paper/film can be easily burnt and thermal degraded after encountering flame attack, thus, in order to obtain desirable GO-based FAS materials, incorporating flame-retardants is needed. However, this may involve the use of organic solvents and lead to compromising mechanical performance. Furthermore, considering the rapid flame spreading speed in real fire accidents [13], it is essential to achieve GO-based FAS materials with ultra-long alarm period and ultra-sensitive and reliable fire alarm response capacity.

To tackle the issues presented above, herein, we design and synthesize the water-soluble multi-amino molecule, named HCPA, via modifying the hexachlorophosphazene (HCCP). Then, the HCPA was used to decorate GO sheets to fabricate GO/HCPA hybrid networks. Based on its six-amine groups in structure and chemical components, the HCPA can serve triple roles including cross-linker, flame retardant and reducing agent. By rationally utilizing HCPA, the optimized GO/HCPA hybrid network shows numerous features such as mechanically flexibility, excellent flame retardancy (keeping structural integrity even after butane torch flame attack), ultra-sensitive fire alarm responses (only ~ 3 and 1 s alarm response times at 300 and 350 °C, respectively) as well as exceptional intumescent effect (more than 40 times increment in thickness), which is superior to most previously reported works. Moreover, based on GO/HCPA hybrid network, the high-performance flame retardant coating with highly adhesive and superhydrophobic properties is designed and applied in rigid PU foam and shows excellent fire-shielding effect (limiting oxygen index (LOI) value of modified foam material can reach ~ 36.5%). Therefore, based on structural characterizations and chemical analysis, the relevant mechanisms, e.g., flame retardancy and intumescent effect, are also be discussed and clarified.

## Experimental

### Materials

Graphite power was supplied by Shanghai Yifan graphite Co., Ltd. (China). Commercial rigid polyurethane (RPU) foam with a density of ~ 150 kg m^−3^ was purchased by Wuxi Kezhao Polyurethane Material Co., Ltd. (China). Other chemicals and reagents including potassium persulfate (K_2_S_2_O_8_) phosphoruspentoxide (P_2_O_5_), potassium permanganate (KMnO_4_), concentrated sulfuric acid (H_2_SO_4_, ≥ 98 wt%) hydrochloric acid (HCl, 35 wt%), hydrogen peroxide (H_2_O_2_, 30 vol%), tetrahydroperfluorodecyltrimethoxy silane (TFTS, > 98%) were supplied from Sinopharm Chemical Reagent Co., Ltd. (China).

### Preparation of GO, HCPA and GO/HCPA Nanocomposite Papers

GO nanosheets were synthesized according to previously reported modified Hummer’s method [38–40]*.* HCPA was synthesized based on a modification method of HCCP, which was introduced in previous work [41]. The detailed synthesis procedure of GO/HCPA papers is as follows: firstly, ~ 4 mg mL^–1^ GO solution was ultrasonic treated for 10 min, then a certain amount of HCPA was dispersed into above GO solution, and the uniform aqueous GO/HCPA solution was obtained after a 2 h magnetic stirring process. Afterward, the resulting mixture suspension was transferred to the mold container and placed in oven at 40 °C for about 12 h. After a slow water evaporation process, the G_x_H_y_ nanocomposite papers were fabricated. For convenience, the GO/HCPA paper was symbolized as G_x_H_y_ paper, where x/y represents the mass ratio of the GO and HCPA components, respectively.

### Preparation of Flame Retardant PU Foam

The preparation of FRPU involves the construction processes of coatings. Specifically, three-layer coatings including Poly (VS-co-HEA) [9], GO/HCPA and TFTS coating, which are responsible for adhesion, flame retardancy and hydrophobicity, respectively, is constructed on to the RPU foam surface. It should be noted that to improve the interfacial adhesion between RPU matrix surface with GO/HCPA coating, ~ 2 mg cm^−2^ content of Poly (VS-co-HEA) coating is firstly applied to the foam surface as a primer by brush-coating (Fig. S12). Another GO/HCPA hybrid coating with different contents of ~ 1–4 mg cm^−2^ was then applied to on the primer surface, which is to play a key role in fire retardancy. Noted that the mass ratio of GO and HCPA is 2:1. To improve the hydrophobicity of the hybrid coating, the sample was further treated with TFTS, achieving a waterproof layer on the resultant foam sample surface. For convenience, the FRPU was symbolized as FRPU-x, where x represents the mass of GO/HCPA coating per square centimeter on surface. For instance, FRPU-2.0 represents the PU foam containing with a mass of 2.0 mg cm^−2^ GO/HCPA coating.

### Characterizations

The detailed characterization methods are provided in Supporting Information.

## Results and Discussion

### Design Principle and Structural Characterizations

**Fig. 1:**
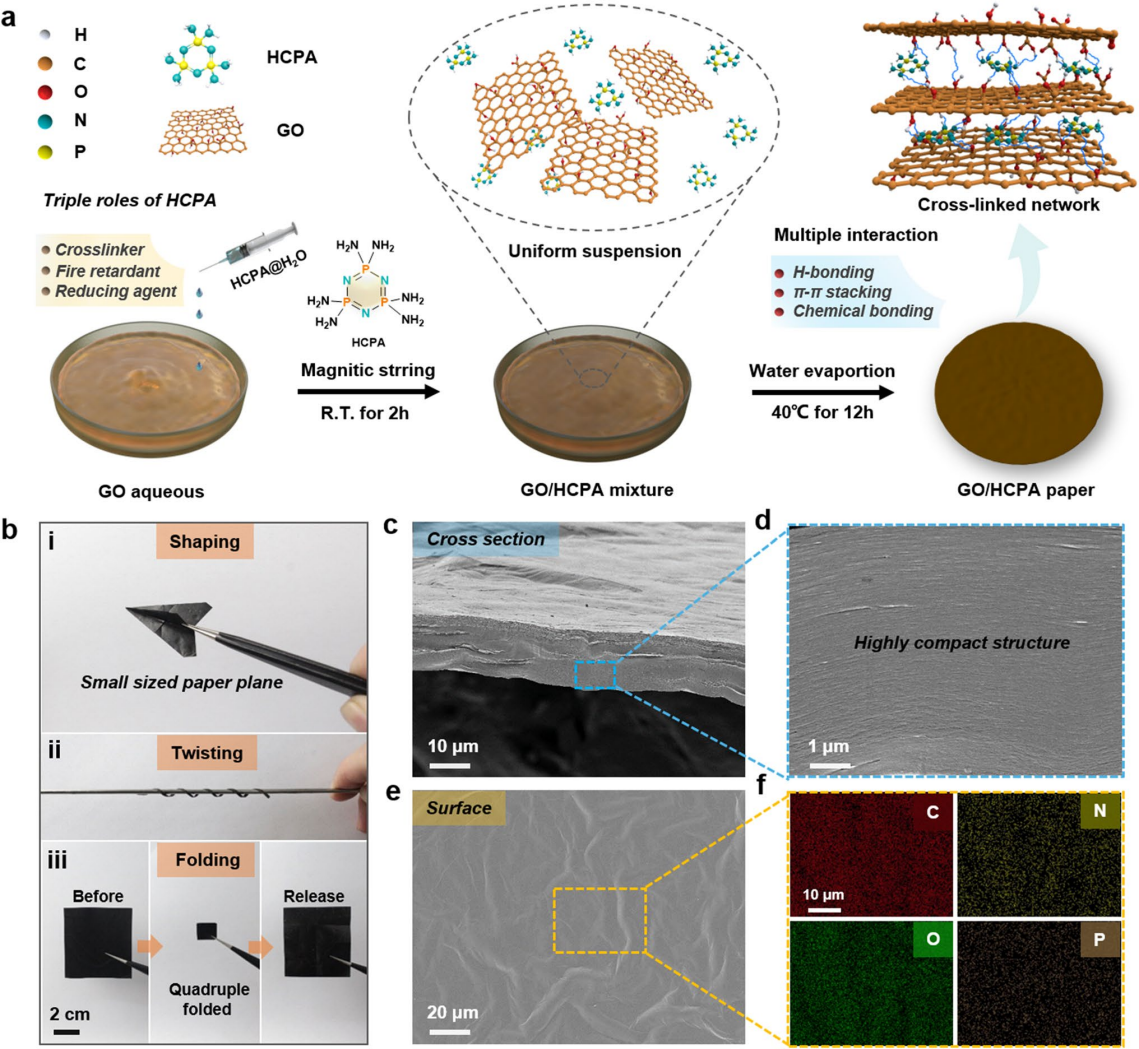
Conceptual design, fabricating process and microstructure. **a** Scheme for preparing the GO/HCPA paper nanocomposites via a simple water evaporation-induced self-assembly strategy. **b** Digital photograph of GO/HCPA paper, the excellent mechanical strength and flexibility can make it withstand shaping, twisting and folding, and no cracks or damages are observed. Typical SEM images of GO/HCPA paper: **c**, **d** cross section and **e**, **f** surface and corresponding EDS mapping images for C, O, N and P

By rationally utilizing the distinctive chemical structure and characteristics of water-soluble HCPA molecules, the multi-functional graphene oxide-based hybrid networks were designed and fabricated via a simple evaporation-induced self-assembly strategy (EISA) (Fig. 1a). Briefly stated, the stable and uniform GO/HCPA aqueous suspension was formed by adding HCPA solution dropwise under continuous stirring. After a low-temperature-induced water evaporation process, the GO/HCPA hybrid networks were obtained. In this study, the HCPA molecules were played triple roles, i.e., cross-linker, flame retardant and reducing agent, thus, achieving high-performance GO/HCPA hybrid networks are expected to apply in fire safety and prevention field. Firstly, with a six-amino group structure, HCPA molecules can attach GO sheets through multiple interactions such as multi-hydrogen bonds and chemical bonds, which can significantly improve the strength of GO cross-linked network. Simultaneously, π–π stacking interactions between GO and HCPA also can be formed due to the hexatomic ring of HCPA molecules, further enhancing interactions between GO sheets. Secondly, as mentioned above multiple interactions between GO and HCPA, the HCPA molecules can provide excellent flame retardancy to GO network due to the high content of P and N atoms, and this involved with multiple flame-retardant mechanisms (i.e., condense and gas flame retardant mechanisms). Thirdly, HCPA molecules can also be employed as reducing agent to promote the thermal-reduction process of GO sheets under high temperature or flame attack conditions, due to the existence of P and N atoms [40, 42, 43]. More importantly, with excellent flame retardancy, such GO/HCPA network is expected to use as ideal fire alarm sensor and fireproof material.

Based on the design of multiple interactions in GO/HCPA hybrid network, as a result, as-prepared GO/HCPA paper shows outstanding mechanical performance, as shown in Fig. 1b, the excellent mechanical strength and flexibility can make it withstand shaping into complex shapes (e.g., small sized paper plane), besides, it can be twisting around thin metal bar (~ 2.5 mm in diameter) and even quadruple folding, and no cracks or damages are observed after release. The micro morphology of GO/HCPA was characterized by SEM, as shown in Fig. 1c–f. Compared to the multilayered micro-structure of pure GO paper (Fig. S1), the GO/HCPA paper shows highly compact cross-sectional structure, and no obvious layered structures are observed, indicating the presence of HCPA molecules enhancing the interactions between GO sheets, and more evidences and analysis will be provided and discussed later. Besides, as expected, the surface of GO/HCPA paper shows a typical wrinkle structure (Fig. 1d), which attribute to the thin thickness of GO sheets (Fig. S2). Moreover, the corresponding EDS mapping images show that the P and N elements are uniformly dispersed on the surface of GO/HCPA paper, further demonstrating the effective and successful HCPA functionalization in the GO/HCPA paper.

### Interaction Characterization and Mechanical Properties

**Fig. 2:**
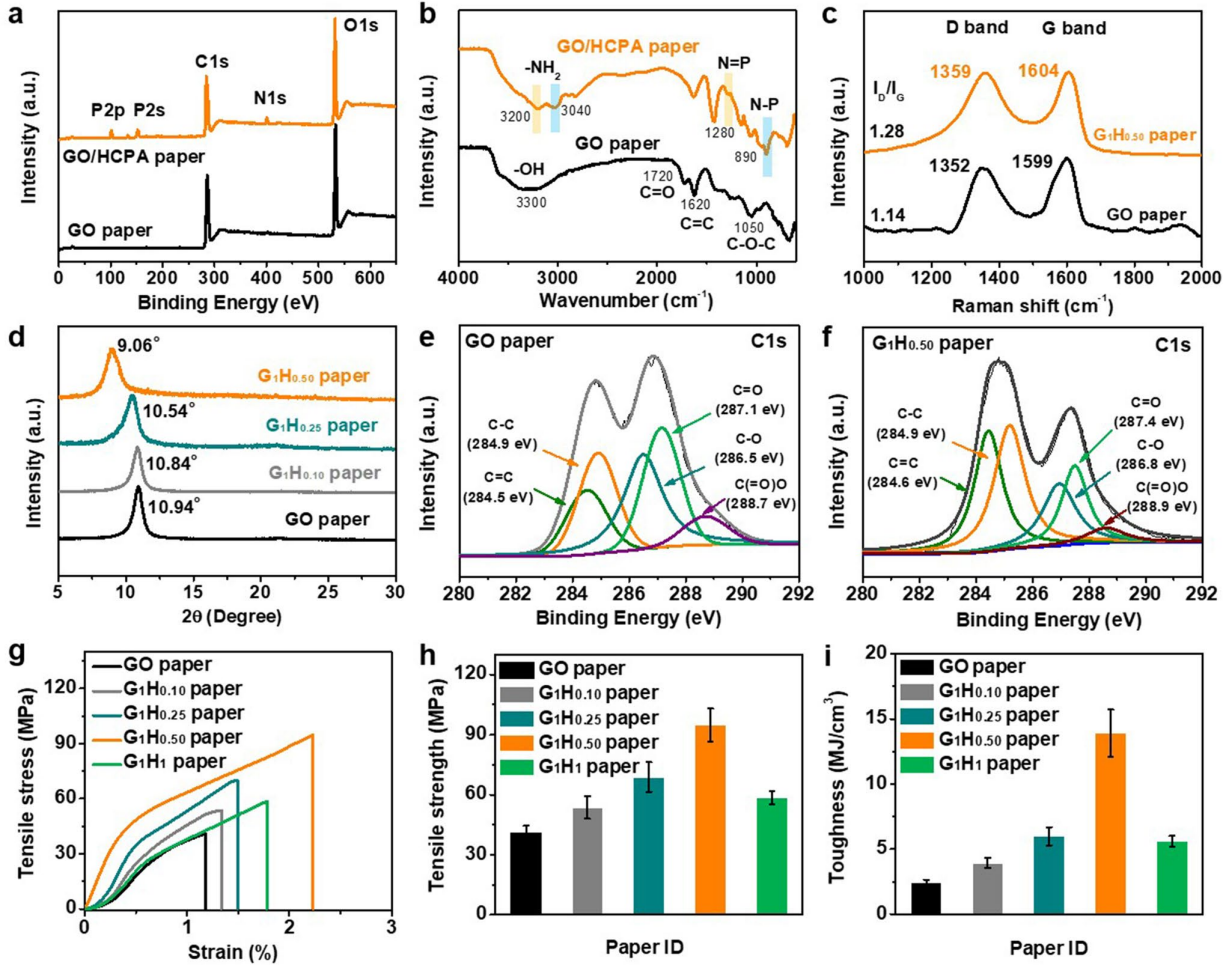
Interaction characterization and mechanical properties. **a** XPS results, **b** FTIR, and **c** Raman spectra of GO paper and G_1_H_0.50_ paper. **d** XRD patterns of various GO/HCPA papers. XPS C 1* s* spectra of **e** GO paper and **f** G_1_H_0.50_ paper. **g** Tensile stress–strain curves, **h** tensile strength and **i** toughness of pure GO paper and various GO/HCPA composite papers, confirming the multi-interaction between GO and HCPA molecules

XPS, FTIR, Raman and XRD spectra were applied to verify the chemical structure and the formation of multiple interactions including hydrogen bond, π–π stacking and chemical bond in the GO/HCPA hybrid network. The XPS survey spectra of GO and GO/HCPA papers are shown in Fig. 2a, compared to pure GO paper, apart from two characteristic peaks of C 1* s* (~ 285 eV) and O 1* s* (~ 533 eV), three new peaks of P 2*p*, P 2* s,* and N 1* s* can be observed, indicating HCPA molecules are attached to the surface of GO sheets [44, 45]. Figure 2b shows FTIR results of GO and HCPA paper; a broad absorption peak at ~ 3300 cm^−1^ for the hydroxyl groups (−OH) can be seen for GO sheets [35, 46]. For GO/HCPA paper, two peaks at 3200 and 3040 cm^−1^ are corresponding to the characteristic absorption of −NH_2_. In addition, it can be also found that the typical peaks at ~ 1280 and ~ 890 cm^−1^, which are assigned to the N = P and N–P, respectively [47]. Typical Raman spectra of GO and GO/HCPA papers are illustrated in Fig. 2c. As we all know, the D peak was related to the disorder degree of the structure and the G peak was attributed to the carbon atoms of graphene possessing a complete *sp*^2^ hybridization [48]. By comparison of the GO paper and GO/HCPA paper, it is clear that with the addition of HCPA molecules, the characteristic D peak and G peak were shifted to higher wavenumber (from 1352 to 1359 cm^−1^ and 1599 to 1604 cm^−1^, respectively), indicating the existence of hydrogen-bond in GO/HCPA network [49]. Further, the *I*_D_ /*I*_G_ ratio value increases from 1.14 for GO paper to 1.28 for GO/HCPA paper, mainly due to the *π–π* stacking between GO sheets and HCPA molecules [33, 50]. XRD was employed to further analyze the interaction mechanism between GO sheets and HCPA molecules. As shown in Fig. 2d, compared with the sharp diffraction peak at ~ 10.94° (interlayer spacing of ~ 0.81 nm) for GO paper, the GO/HCPA papers show large interlayer spacing after the presence of HCPA molecules, and the interlayer spacing value can further increase with a higher HCPA content. More specifically, ~ 10.84° (~ 0.82 nm) for G_1_H_0.10_, ~ 10.54° (~ 0.84 nm) for G_1_H_0.25_ and ~ 9.06° (~ 0.98 nm) for G_1_H_0.50_, respectively, indicating the HCPA molecules among to the GO sheets [51]. The interlayer spacing increases of GO/HCPA papers with increasing the HCPA contents are attributed to the effective HCPA molecules intercalation and the formation of multi-interactions e.g., hydrogen bonding and π–π stacking interactions among GO sheets [52]. Moreover, the XPS C 1* s* spectra of GO and GO/HCPA papers can verify the existence of chemical bond in GO/HCPA network as shown in Fig. 2e. Compared to GO paper, the intensity of peaks of oxygen-containing groups was obviously decreased. Furthermore, the peaks of C − O, C = O and C(= O)–O groups in the GO/HCPA paper shifted to higher binding energy (from 286.5, 287.1, and 288.7 eV to 286.8, 287.4, and 288.9 eV, respectively), further demonstrating the existence of hydrogen-bonding and π–π stacking in GO/HCPA network (Fig. 2f). Furthermore, the appearance of peak of O = C–N in XPS N 1* s* spectrum of GO/HCPA paper verified that the amino groups of HCPA molecules were successfully bonded on the graphene oxide surface (Fig. S3). The above results indicate the successful formation of multi-interaction in GO/HCPA hybrid network, thereby achieving a significant mechanical enhancement effect on the GO/HCPA papers.

The mechanical properties of GO paper and various GO/HCPA papers were investigated and the related results as shown in Fig. 2g, i. Figure 2g displays the strain–stress curves of paper samples; obviously, the tensile strength of GO/HCCP paper was greatly improved by introducing HCPA molecules to GO network, and the value is strongly related to the HCPA content, more specifically, ~ 54 MPa for G_1_H_0.10_ paper, ~ 69 MPa for G_1_H_0.25_ paper and ~ 95 MPa for G_1_H_0.50_ paper, respectively, compared to pure GO paper of ~ 41 MPa (Fig. 2h). Satisfyingly, along with the increases in tensile strength, the elongation at break of GO/HCPA papers was also gradually increased. More importantly, similar significant improvement in toughness can be also found in Fig. 2i. It is worth noting that with a higher content of HCPA, the GO/HCPA paper shows obvious decrease both in elongation at break, tensile strength, and toughness in resultant G_1_H_1_ paper sample, this phenomenon is consistent with previously reported result. Comparatively, the G_1_H_0.50_ paper shows desirable integration of strength and toughness of ~ 95 MPa and ~ 14 MJ m^−2^, respectively, which are 2.3 times and 5.6-times higher than that of pure GO paper. The detailed mechanical properties of other GO/HCPA papers are listed in Table S1. In addition, our as-prepared GO/HCPA paper also shows distinct advantages over other previously reported similar GO-based paper systems in terms of mechanical strength (Table S2). Summarizing and analyzing above results, it is reasonable to confirm that the multiple interactions were formed in GO/HCPA hybrid network. Firstly, for pure GO paper, once being stretching, due to relatively weak bonding interaction between the GO sheets, the GO sheets begin to slide over each other and the crack appears, and immediate fracture will consequently occur, thus leading to undesirable elongation at break and tensile strength [53]. After addition of HCPA molecules, multi-interactions, e.g., hydrogen bond, π–π stacking and chemical bond, can be formed in GO/HCPA hybrid network based on abundant hydroxyl groups in GO sheets surface and multi-amino group of HCPA molecular structure, and mechanical strength of cross-linked GO network can be enhanced with increasing HCPA content, thus leading to the improved mechanical properties of GO/HCPA papers [41, 49]. However, once beyond appropriate content of HCPA, e.g., the mass ration ratio of 1:1 (GO: HCPA), bonding interaction among GO sheets becomes weak, thus further results them to slide over from initial position under a loading force which is much lower than the strength of the GO/HCPA papers with low content of HCPA [33], the obvious cracks in layered structure of G_1_H_1_ paper can well demonstrate this (Fig. S4).

### Flame-Retardant and Intumescent Performances and Mechanism Analysis

**Fig. 3:**
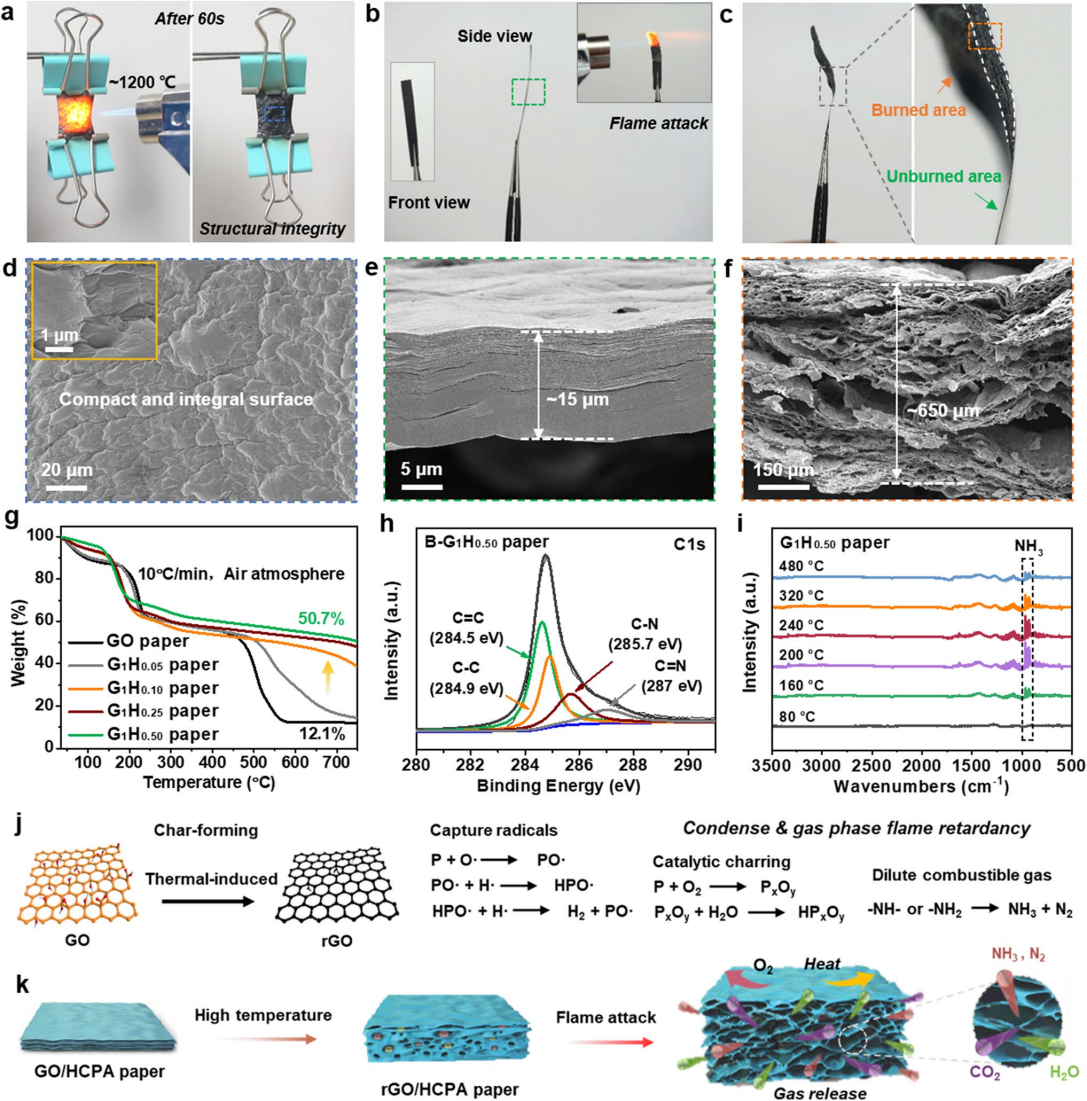
Flame retardant performance and its mechanisms. **a**–**c** Digital photographs and **d**–**f** SEM images of the G_1_H_0.50_ paper before/after being exposed to the butane blowtorch flame (~ 1200 ℃) for 1 min, the almost unchanged original shape, structural integrity, and obvious increase in thickness, showing excellent flame retardancy and thermal stability, exceptional intumescent effect of the GO/HCPA paper. **g** TGA results of various GO/HCPA papers. **h** XPS C 1* s* spectra of G_1_H_0.50_ paper after being burned. **i** TG-IR results of G_1_H_0.50_ paper. **j** Multiple flame-retardant mechanism, and **k** intumescent process of the GO/HCPA paper under high temperature and flame attack conditions

To investigate the flame retardancy and flame-induced self-intumescent effects, the GO/HCPA papers were treated under different high-temperature flames conditions, i.e., alcohol lamp flame (~ 500 °C) and butane torch flame (~ 1200 °C). As shown in Fig. S5 and Movie S1, once being exposed to the alcohol lamp flame, the pure GO paper displays rapid decomposition behavior and can be almost completely burned out within only ~ 90 s, which is well consistent with previously reported results [35, 54]. Comparatively, G_1_H_0.10_ paper shows improved flame retardancy, besides, a higher content of HCPA produces more excellent flame retardancy in G_1_H_0.50_ paper. More importantly, after being treated with a higher temperature (~ 60 s butane torch flame attack), almost unchanged shape and structural integrity can also be maintained even being loaded with a metal clip after (Fig. 3a and Movie S2). Furthermore, due to the high nitrogen content of HCPA molecules in GO/HCPA hybrid network, amounts of nonflammable gases, e.g., NH_3_, N_2_, H_2_O and CO_2_, would be released from GO layer space when being exposed to flame attack, thus producing an exceptional intumescent effect [44, 55]. As shown in Fig. 3b, c, obvious increased thickness can be observed by comparing the thickness of burned area and unburned area of G_1_H_0.50_ paper, indicating the self-intumescent behavior. Corresponding SEM images can further confirm this, Fig. 3d presents the surface SEM images of the G_1_H_0.50_ paper after being burned. Obviously, compared to loosen and damaged surface of pure GO paper (Fig. S6), G_1_H_0.50_ paper displays compact and integral surface, and no cracks can be found even in high magnification SEM image (Fig. 3d inset). Interestingly, the surface also exhibits wrinkled and ridged morphology, which is caused by gases release from GO/HCPA network. In addition, due to gases release, the exceptional flame/high-temperature-induced intumescent behavior occurred, and corresponding structural evolution was more clearly shown in Fig. 3e, f. As seen, the G_1_H_0.50_ paper had a parallelly ordered and compact layered structure, which provides the GO/HCPA paper with outstanding flexibility and high mechanical strength (Fig. 1b). Upon being flame attacked, numerous small pores were introduced between the parallel rGO layers and accompanied with a significant increase in thickness, resulting in a well-defined aerogel-like porous structure (Fig. 3d). It is worth noting that the rGO sheets were not individually separated but partially glued to each other to form a continuous cellular structure based on intumescent effect. As a result, the thickness of G_1_H_0.50_ paper was increased from ~ 15 μm (Fig. 3e) to ~ 650 μm, which is beyond 40 times higher than that of original state. Moreover, although the mechanical strength of rGO/HCPA paper is weak after flame attack, it can still display a certain extent of structural stability and mechanical bendability and can be bent beyond 90° without fracture due to its continuous cellular structure (Fig. S7), this is important to real fire alarm application (be discussed later).

In order to compare the thermal stability of pure GO paper and GO/HCPA papers modified with HCPA molecules, TGA measurements were also taken and corresponding TGA curves of various paper samples are shown in Fig. 3g. Clearly, all paper samples were started to lose weight below 100 °C, which was attributed to the volatilization of stored water in the GO network. For pure GO paper, two obvious thermal-degradation stages at 180–220 °C and 450–550 °C can be observed, which are corresponding thermal pyrolysis/decomposition of oxygen-containing groups on GO sheets and thermal degradation of the graphitic framework, respectively [46]. By contrast, GO/HCPA papers show obvious improved thermal stability, and with increasing HCPA content, more excellent thermal stability can be achieved, especially for the temperature range of 500–750 °C. More importantly, with presence of HCPA, the residue mass of papers was significantly increased: ~ 38.6% for G_1_H_0.10_ paper, ~ 47.9% for G_1_H_0.25_ paper, and ~ 50.7% for G_1_H_0.50_ paper, respectively, compared to ~ 12.1% for pure GO paper. In addition, in order to visually compare the structural stability of pure GO paper and GO/HCPA paper in real high-temperature scene, the paper samples were treated under high temperature of ~ 300 °C for 10 min. Clearly, GO paper displayed damaged structure, while almost unchanged size and integral structure can be kept in G_1_H_0.50_ paper (Fig. S8), this result is well consistent with the TGA results.

Based on the above results and analysis, the mechanisms about flame retardancy and intumescent behavior are proposed as shown in Fig. 3j, k. The GO/HCPA paper is composed of multiple flame-retardant systems including graphene oxide, phosphorus atom, nitrogen atom/amino functional groups, which act as the carbon source, acid source and gas source of the GO/HCPA system, respectively, during combustion process. Thus, a multiple-flame retardant mechanism of condensed phase flame retardancy and gas phase flame retardancy is produced. Once being burned, graphene oxide will transform to reduced graphene oxide (char layer) and can act as physical barrier to restrict the external oxygen and heat attack [56]. Besides, phosphorus atom will capture free radicals (e.g., O· and H· radicals) in the gas phase, which can reduce the amount of combustible products and avoid the spread of combustion reactions [47]. Meanwhile, P_x_O_y_ compounds will be formed based on the reaction of phosphorus and oxygen, and these P_x_O_y_ compounds will further generate phosphoric acids with H_2_O, which can promote the formation of surface protective char layers, thus obtaining a condensed-phase flame retardancy [57]. As a result, P and N hybrid elements were doped in the rGO network (Fig. S9), thus resulting in excellent flame retardancy and high thermal stability. Indeed, the XPS C 1* s* and P 2*p* results of GO/HCPA paper after burned can well demonstrate this (Figs. 3h and S9). For instance, compared to GO/HCPA paper, in XPS C 1* s* spectrum of GO/HCPA paper after being burned, the peaks of C − O, C = O, and C(= O)–O groups disappeared, indicating the decomposition of oxygen containing groups. Besides, the appearance of peaks of C − N and C = N at 285.7 and 287 eV, respectively, verified that N atoms were doped in rGO/HCPA network (Fig. 3h). Similarly, P doped phenomenon also can confirm based on XPS P 2*p* spectrum (Fig. S10). On the other hand, the multi-amino group of HCPA molecules would decompose into nonflammable gases (e.g., NH_3_ and N_2_) (Fig. 3i), which will dilute the concentration of combustible gases and further inhibit the combustion behavior. More importantly, along with other gaseous products (e.g., CO_2_ and H_2_O derived from the thermal degradation of oxygen-containing groups on GO sheets), such nonflammable gases blew up the compact GO layers to form aerogel-like porous intumescent network. Considering its excellent flame retardancy, high thermal stability and possible promoting thermal reduction effect on GO sheets due to P and N doping. Such GO/HCPA hybrid networks may possess desirable fire warning function. Therefore, their fire early warning response behaviors under high temperature and flame attack conditions were systematically investigated.

### Fire Early Warning Response Performance

**Fig. 4:**
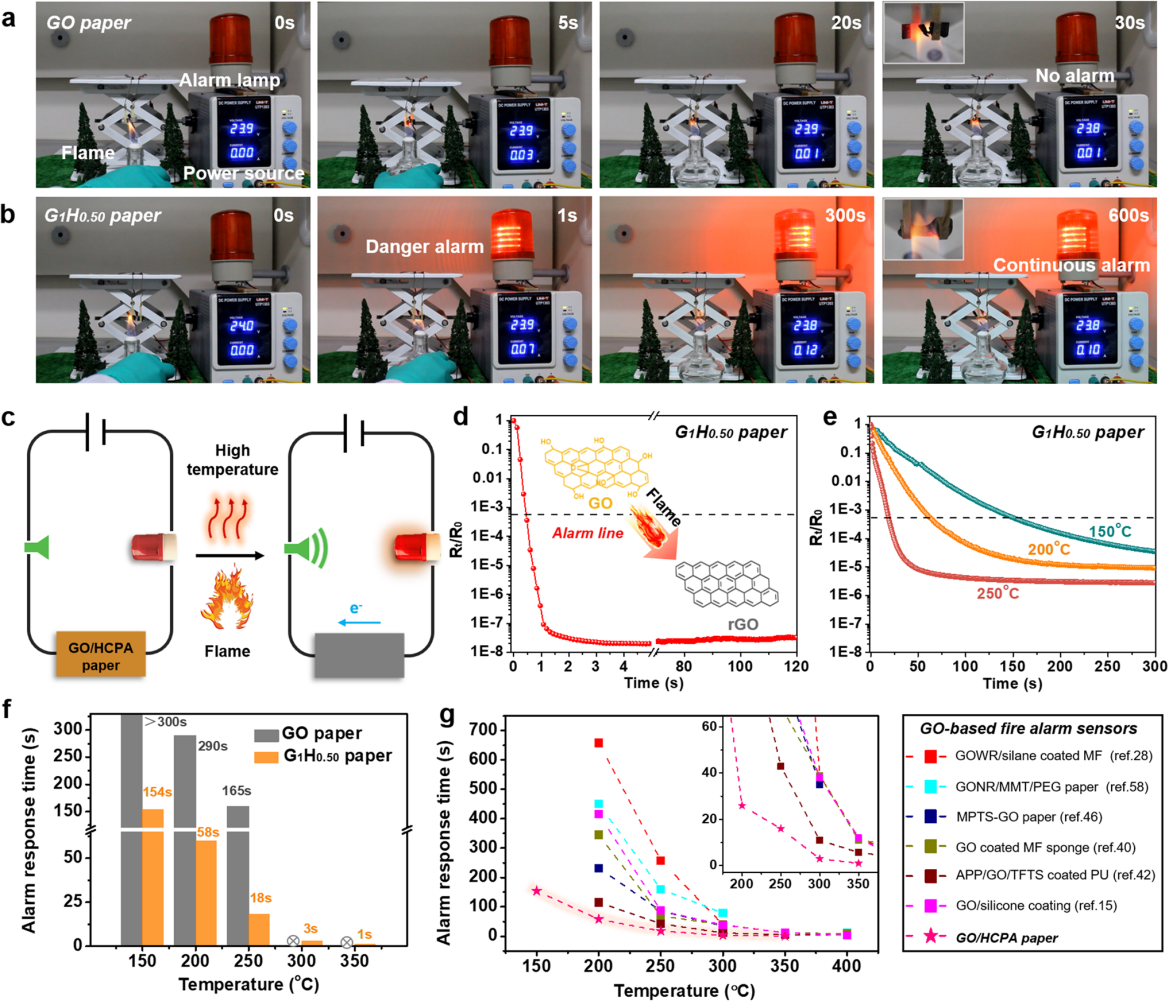
Flame detecting and early alarm response performance. Photographs of flame detection processes of **a** GO paper and **b** G_1_H_0.50_ paper; **c** schematic illustration of fire alarm sensor based on GO/HCPA paper under flame attack or high-temperature condition. Electrical resistance transition behaviors of the G_1_H_0.50_ paper under **d** various environmental temperatures and **e** flame attack, showing rapid fire early warning alarm response below the ignition temperature of most combustible materials. **f** Alarm responsive time of pure GO paper and G_1_H_0.50_ paper under different high-temperature conditions from 150 to 350 °C. **g** Comparison of flame detecting warning alarm response time of the G_1_H_0.50_ paper with other previous reported results

Figure 4c shows the schematic of construction and working mechanism of fire warning system based on the GO/HCPA hybrid network. Once encountered the flame or high-temperature conditions, the GO sheets will be thermally reduced and an electrically conductive path can be formed [15, 33], thus triggering the alarm lamp. To evaluate and compare the practical fire alarm response behaviors of the pure GO paper and GO/HCPA paper, a comparative study was conducted and corresponding flame detection and warning processes are shown in Fig. 4a, b and Movie S3. As seen, when exposed to the ethanol lamp flame, the pure GO paper was quickly thermal decomposed due to its undesirable flame retardancy and thermal stability, and although DC low voltage power supply indicates changed current value, the alarm lamp was not triggered until GO paper was burned out (Fig. 4a). This result is consistent with our previous reported works [51]. Comparatively, for the G_1_H_0.50_ paper, the danger alarm signal can be sent out within ~ 1 s when attacked by flame attack, more importantly, due to excellent flame retardancy and thermal stability, the signal can be well maintained during flame combustion process for beyond 600 s (Fig. 4b). A reliable and long warning period of beyond 600 s can provide people with enough time to tackle fire hazards. Besides, in order to obtain its accurate fire alarm response times, the real-time electrical resistance was monitored and alarm line was also defined based on electrical resistance transition, the danger alarm can be triggered after beyond ~ three orders of magnitude decrease in electrical resistance (corresponding real-time electrical resistance is around 30–50 kΩ). The electrical resistance transition curve of G_1_H_0.50_ paper under flame attack condition is shown in Fig. 4d. Clearly, the rapid electrical transition that occurred and corresponding electrical resistance change of over three orders of magnitude can be completed within only 0.6 s, which is consistent with its fire warning behavior in practical test. (Fig. 4b). In addition, for most flammable materials such as polymeric materials and natural wood, there exists rapid increasing temperature process before materials ignited [43], and the high-temperature warning function is particularly meaningful for reducing or avoiding possible life and property loss. Therefore, the high-temperature warning response behaviors and corresponding electrical resistance changes of G_1_H_0.50_ paper under different high-temperature conditions were also conducted and monitored as shown in Figs. 4e, S11 and Movie S4. As expected, with increasing the treating temperature, more quick electrical resistance transitions can achieve (Fig. 4d), and a desirable high-temperature response time of ~ 150 s can obtain even under 150 °C condition. By contrast, the electrical resistance of pure GO paper was almost unchanged within 300 s under the same condition according to our previous work [51, 58]. This is because the thermal reduction process of GO sheets was hard to proceed under such temperature condition. In addition, the results of the high-temperature warning response of G_1_H_0.50_ paper in real tests are well consistent with corresponding electrical resistance transitions behaviors, which are shown in Movie S4. The detailed high-temperature warning response time for GO paper and G_1_H_0.50_ paper is summarized and shown in Fig. 4f. Obviously, compared to GO paper, G_1_H_0.50_ paper shows satisfying wide temperature detecting range and rapidly fire alarm response at a fixed temperature, such as 18 s for 250 °C, ~ 3 s for 300 °C and ~ 1 s for 350 °C, respectively. Previous studies indicated that the temperature-induced electrical resistance transition behaviors of GO-based fire warning sensor systems are largely dependent on the thermal reduction process of GO network [51]. Based on this, various strategies, e.g., introducing reducing agent [51, 59] or thermal conductive filler [44, 60] and modifying GO sheets [61, 62], were adopted to shorten the thermal reduction process of GO. In this work, by introducing HCPA molecules to GO network, the existence of P and N doping reactions in rGO network during the flame or high-temperature conditions is the main reason obtaining sensitive early fire warning responses, and the relevant mechanism was well investigated and discussed in previous works [40, 42]. Moreover, based on above results and analysis, the fire early warning response times of our as-prepared sensor and other reported GO-based FAS systems were summarized and compared and are shown in Fig. 4e. By comparison, it is obvious that our GO/HCPA paper sample shows wide-temperature detecting capability and ultra-sensitive fire alarm response behaviors, which is superior to other GO-based flame detection/early-warning sensors.

### Fire Safety Application of Hybrid Nanocoating

**Fig. 5:**
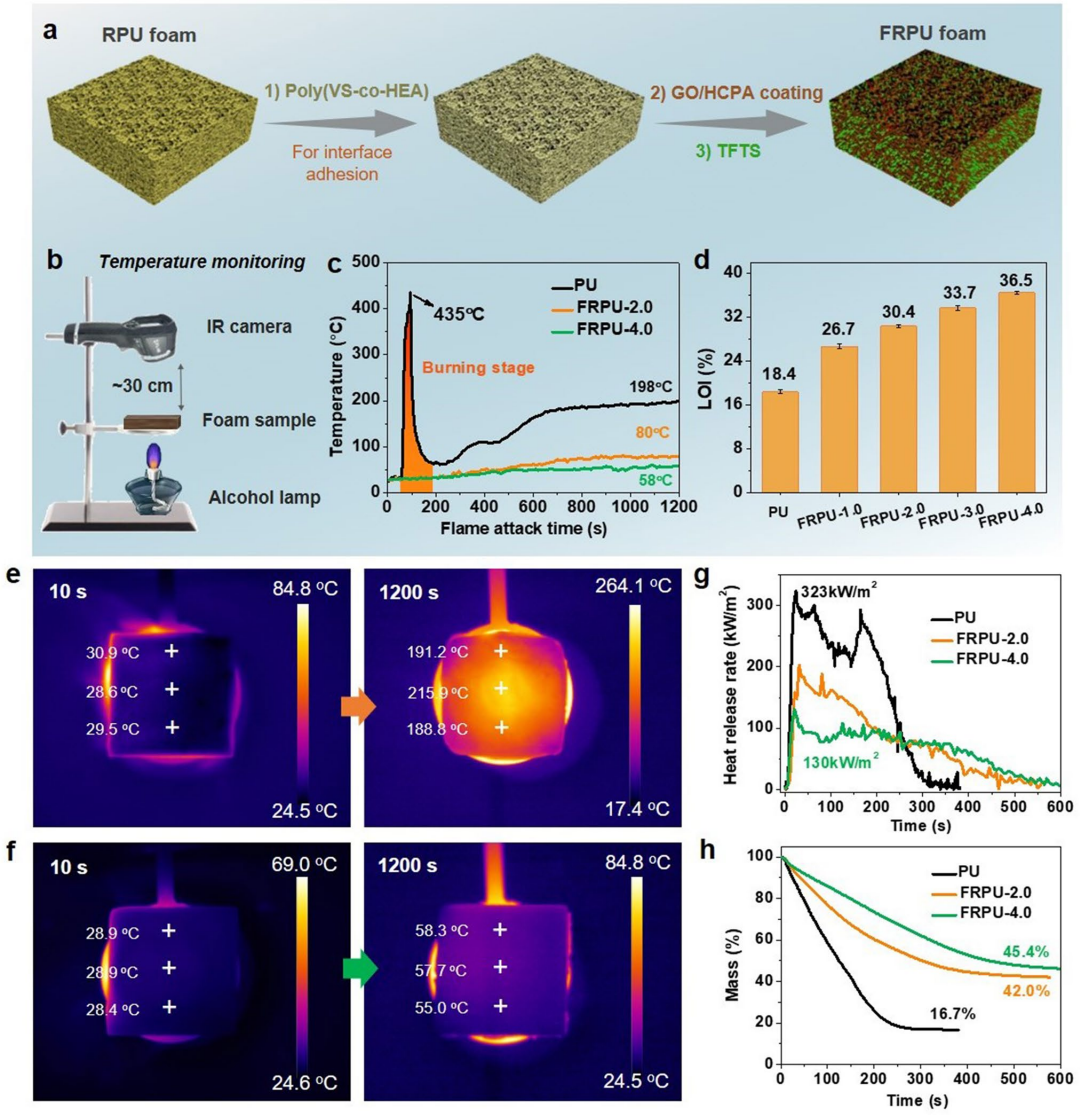
Flame-retardant coatings application to RPU foam. **a** Schematic illustration for the fabrication process of flame-retardant RPU foam (FRPU). **b** Homemade setup for determining real-time temperature changes of PU foam samples during combustion process and. **c** Top-surface temperature of PU and FRPU samples as a function of flame attack time. **d** LOI values of various PU foam samples. Infrared images of **e** PU foam and **f** FRPU-4.0 exposed to the flame attack for 10 and 1200 s. **g** HRR and **h** mass as a function of time of various foam samples

**Fig. 6:**
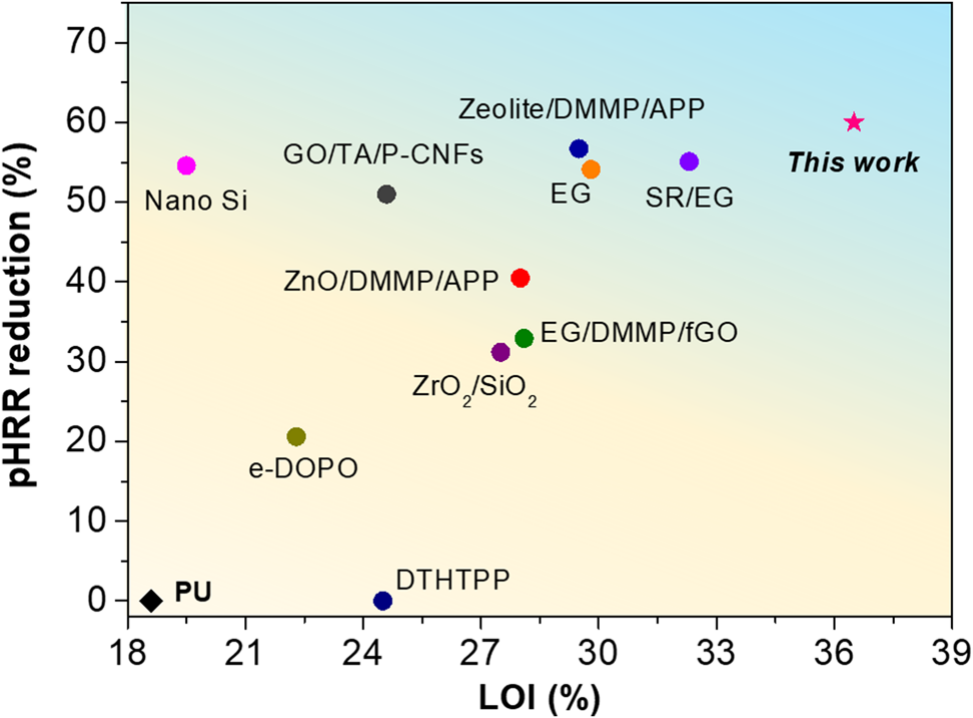
Flame retardancy comparison (pHRR reduction and LOI value) of as-prepared FRPU with other previously reported flame-retardant PU foam material systems

Rigid polyurethane foam (RPF) as one of the most attractive commercial polymer foams has been commonly used for building energy saving due to their numerous features, including light-weight, low-cost, thermal-isolation, and outstanding mechanical strength [63]. However, porous structure and high organic components of RPF render it intrinsic flammability, once ignited, RPUF may pose potential fire hazards. The above-mentioned results and discussions indicate that the GO/HCPA hybrid networks can be applied as an effective flame retardant nanocoating for combustible RPF due to its outstanding mechanical flexibility, excellent flame retardancy and exceptional intumescent effect. Herein, the surface coating method was adopted to prepare flame-retardant RPF materials. As shown in Fig. 5a, apart from constructing flame retardant GO/HCPA coating, in order to achieve a desirable interfacial adhesion between foam matrix and coating, the polyhydroxy copolymers poly(VS-co-HEA) was employed. Besides, a hydrophobic coating based on TFTS was also introduced to improve the surface hydrophobicity of foam. As a result, the coating with good interfacial adhesion and superhydrophobic surface was obtained as shown in Figs. S13 and S14. The surface chemical analysis and morphology of FRPU surface also were systematically investigated, and the related results can be obtained from Figs. S15-S18. In addition, considering its real application scene, the frictional resistance property of coating is also vital, thus, the test was also conducted. Satisfyingly, the test result indicates that such flame-retardant coating exhibits a certain extent of wear resistance property (Fig. S19). In order to visually evaluate and compare the flame retardant performance of untreated RPU and FRPU with various FR coating, the burning tests were conducted, and a homemade setup is constructed to monitor the top surface temperature of foam samples during the burning process (Fig. 5b). As expected, due to high flammability, once pure RPU being ignited, the fire was rapidly spread to whole sample. The surface average temperature (SAT) of untreated RPU increased sharply (Movie S5) and reached the peak of ~ 435 °C within 1 min (Fig. 5c). By contrast, the FRPU-2.0 and FRPU-4.0 sample shows a slow increase in the surface temperature rising and their SAT values are much lower than that of the untreated one during whole burning process, which is attributed to their excellent flame retardancy. More specifically, their SAT values at 20 min are of ~ 80 °C for FRPU-2.0 and ~ 58 °C for FRPU-4.0, respectively, compared to ~ 198 °C for the untreated one. Corresponding SAT changes of pure RPU foam and FRPU-4.0 can be reflected by infrared images in Fig. 5e, f. Moreover, after 30 min flame attack, foam samples showed different morphologies. For pure RPU foam, its original shape was severely destroyed after undergoing burning and thermal ablation processes, thus leaving a broken char residue with hollow shell structure (Fig. S20). Comparatively, because of the fire protection of the FR coatings, all treated RPU foam produces intumescent char layer on the backside (burned zone), and its top side preserved its original structure and size. Further, the height of char zone of sample residual displays a certain decrease with FR coatings, indicating a higher content of FR coating can produce a more desirable fire-shielding performance. The results of LOI of foam samples (Fig. 5d) and their combustion behaviors at a fixed oxygen concentration (Figs. S21-S22 and Movie S5) can further demonstrate this. Flame retardancy of the FRPU was further evaluated by cone calorimetry test. As presented in Fig. 5g and Table S3, the peak of heat release rate (pHRR) of pure RPU foam is high up to ~ 323 kW m^−2^, with a ~ 4.0 mg cm^−2^ loading of FR coating, the value can decrease to ~ 130 kW m^−2^ (~ 60% decrease in pHRR). Therefore, the total heat release is also reduced effectively (Fig. S23). More importantly, char residual mass of foam samples was significantly increased with the presence of FR coating, 42.0% for FRPU-2.0 and 45.4% for FRPU-4.0, compared with ~ 16.7% for pure RPU foam (Figs. 5h and S24). Therefore, the corresponding UL94 flame-retardant grade of foam samples was also summarized and shown in Table S4. In order to highlight the superiority of the as-designed triple-coating to previously reported works [63–72], both the pHRR reduction and LOI values of as-designed FRPU composites and other FRPU foam systems are plotted and compared in Fig. 6 and the detailed information, e.g., flame retardant systems, processing method, loading and flame retardant properties, are summarized in Table 1. Overall, in terms of fire-shielding performance our as-prepared FR coatings show advantages over other flame-retardant coatings or additive flame-retardant fillers, as a relatively low loading of FR coating can produce satisfying fire safety performance. Indeed, besides rigid PU foam material, such FR coating also shows excellent potential application in other flammable material systems such as rigid natural wood and flexible polymer foam (Fig. S25). Based on the above results and discussions, therefore, besides ultra-sensitive FAS material, such GO/HCPA hybrid network can also be applied as desirable fireproof coating for fire safety and prevention application.

**Table 1:** Comparison of flame-retardant performance of our as-prepared FRPU and other PU foam material systems

Flame retardant systems^a^	Processing method	Loading (wt% or mg cm^−2^)	LOI value (%)	pHRR (kW m^−2^) (Composite/Control)	Residue mass (wt%)	Refs
TDHTPP	Addition	5 wt%	NM	260/265 (− 1.9%)	25.5	[67]
		10	NM	266/265 (− ~ 0%)	28.5	
		15	24.5	266/265 (− ~ 0%)	29.5	
ZnO/DMMP/APP	Addition	5/8/8 wt%	28.0	125/210 (− 40.5%)	NM	[69]
MMT/DMMP/APP		5/8/8	28.5	119/210 (− 43.3%)	NM	
Zeolite/DMMP/APP		5/8/8	29.5	91/210 (− 56.7%)	NM	
EG/DMMP	Addition	7.5/2.5 wt%	26.5	199/272 (− 26.8%)	NM	[68]
EG/DMMP/GO		7.5/2.5/0.25	27.5	186/272 (− 33.1%)	NM	
EG/DMMP/fGO		7.5/2.5/0.25	28.1	182/272 (− 32.9%)	17.6	
e-DOPE	Addition	3.78 wt%	22.3	271/281 (− 3.6%)	16.6	[65]
		8.80	22.2	228/281 (− 18.9%)	10.7	
		12.22	22.3	223/281 (− 20.6%)	4.0	
EG-1	Addition	10 wt%	29.8	111/220 (− 49.5%)	46	[70]
EG-2		10	29.8	113/220 (− 48.6%)	47	
EG-3		10	29.8	101/220 (− 54.1%)	50	
ZrO_2_/SiO_2_	Addition	1.0 wt%	27.2	899/1130 (− 20.4%)	NM	[66]
		2.0	27.3	828 (− 26.7%)	NM	
		3.0	27.5	777 (− 31.2%)	NM	
SR	Coating	7.1 mg cm^−2^	22.8	230/245 (− 6.1%)	26.7	[63]
SR/EG		7.1	32.3	110/245 (− 55.1%)	38.6	
Alginate/Clay	Coating	200 μm	38	223/323 (− 31.0%)	9.4	[71]
		700	60	220 (− 35.0%)	19.7	
Nano-Si	Coating	3.7 wt%	18	533/560 (− 4.8%)	0.3	[64]
		7.0	19.0	282/ (− 49.6%)	16.5	
		33.9	19.5	254 (− 54.6%)	24.9	
GO/TA/P-CNFs	Coating	10 wt%	20.0	164/318 (− 48.4%)	26	[72]
		20	22.8	NM	NM	
		30	24.6	154 (− 51.6%)	30	
*PVS/GO/HCPA*	*Coating*	*2.0/1.0* mg cm^−*2*^	*26.7*	*NM*	*NM*	*This work*
		*2.0/2.0*	*33.7*	*203/323 (*− *37.2%)*	*42.0*	
		*2.0/4.0*	*36.5*	*130/323 (*− *60.0%)*	*45.4*	

^a^TDHTPP: 2,4,6-triphosphoric acid diethyl ester hydroxymethyl phenoxy-phosphonate magnesium hydroxide; ZnO: zinc oxide; DMMP: dimethyl methyl phosphonate; APP: ammonium polyphosphate; MMT: montmorillonite; EG: expandable graphite; GO: graphene oxide; fGO: functionalized GO; e-DOPE: epoxidized 9,10-dihydro-9-oxa-10-phosphaphenanthrene-10-oxide; EG1: volume expansion of 250 cm^3^ g^**−1**^ and 300 µm particle size; EG2: expansion volume of 350 cm^3^ g^**−1**^ and 300 µm particle size; EG3: expansion volume of 350 cm^3^ g^**−1**^ and 500 µm particle size; SR: silicon resin; TA: tannic acid; P-CNFs: phosphorylated-cellulose nanofibrils; PVS: poly(VS-co-HEA)

^b^NM: not mentioned

## Conclusion

In this work, water-soluble multi-amino molecule HCPA was employed to achieve GO-based hybrid networks with exceptional flame resistance and ultra-fast fire warning response. By designing and utilizing the triple roles of HCPA, i.e., cross-linker, flame retardant and reducing agent, the GO/HCPA hybrid networks were prepared by a facile EISA method. Because of the formation of multi-interactions including hydrogen-bond, π–π stacking, and chemical bond in GO network, the optimized GO/HCPA network exhibit significant improvement in mechanical properties, e.g., tensile strength and toughness reach ~ 95 MPa and ~ 14 MJ cm^−3^, ~ 2.3 and ~ 5.7 times higher than that of the pure GO paper, respectively. Additionally, the resultant GO/HCPA hybrid networks present excellent high-temperature resistance and exceptional intumescent effect. More importantly, the thermal reduction process of GO network was significantly promoted with the presence of HCPA, and the realized high-temperature alarm responses (e.g., 300 °C for 3 s and 350 °C for 1 s) are the shortest times reported for GO-based FAS so far. Furthermore, based on GO/HCPA hybrid network, the high-performance hybrid coating onto the rigid PU foam is constructed and applied for achieving good adhesion, excellent fire-shielding features and surface superhydrophilicity. Clearly, owing to numerous features, e.g., mechanical flexibility, excellent flame retardancy, ultra-sensitive fire early alarm response as well as exceptional intumescent effect, such GO/HCPA hybrid networks show promising fire safety and prevention applications.

## Supplementary Information

Below is the link to the electronic supplementary material.

Supplementary file1 (PDF 1545 KB)

Supplementary file2 (MP4 10632 KB)

Supplementary file3 (MP4 10045 KB)

Supplementary file4 (MP4 11134 KB)

Supplementary file5 (MP4 11064 KB)

Supplementary file6 (MP4 8597 KB)
