# Supplementary material for: Fire Intumescent, High-Temperature Resistant, Mechanically Flexible Graphene Oxide Network for Exceptional Fire Shielding and Ultra-Fast Fire Warning
Source: Nanomicro Lett. 2022 Apr 6;14:92. doi: 10.1007/s40820-022-00837-1 (PMC8986961; doi:10.1007/s40820-022-00837-1)
Supplement: Supplementary file 1 — Supplementary file1 (PDF 1545 KB) [file 40820_2022_837_MOESM1_ESM.pdf]

Supporting Information for

Fire Intumescent, High-Temperature Resistant, Mechanically Flexible Graphene Oxide Network for Exceptional Fire Shielding and Ultra-Fast Fire Warning

Cheng-Fei Cao[1], Bin Yu[2,*], Zuan-Yu Chen[3], Yong-Xiang Qu[3], Yu-Tong Li[3], Yong-Qian Shi[4], Zhe-Wen Ma[5], Feng-Na Sun[3], Qing-Hua Pan[3], Long-Cheng Tang[3], Pingan Song[1], Hao Wang[1,*]

[1]Centre for Future Materials, University of Southern Queensland, Springfield Central, 4300, Australia

[2]State Key Laboratory of Fire Science, University of Science and Technology of China, Hefei 230026, P. R. China

[3]College of Material, Chemistry and Chemical Engineering, Key Laboratory of Organosilicon Chemistry and Material Technology of MoE, Hangzhou Normal University, Hangzhou 311121, P. R. China

[4]College of Environment and Resources, Fuzhou University, Fuzhou 350116, P. R. China

[5]School of Engineering, Zhejiang A & F University, Hangzhou 311300, P. R. China

[*]Corresponding authors. E-mail: yubin@ustc.edu.cn (B. Yu), Hao.Wang@usq.edu.au (H. Wang)

S1 Experimental Section

S1.1 Characterizations

FTIR spectra of various paper samples were performed on a FTIR spectroscopy (Antaris, Nicolet 7000) in the range of 600 to 4000 cm^{-1}. XPS spectra of samples were used to identify the elemental compositions with a photoelectron spectrometer (VG Scientific ESCALab 220I-XL). XRD results were recorded on a D/Max 2550 V X-ray diffractor (Rigaku, Japan). The morphologies of the samples were characterized by scanning electron microscopy (SEM) (Sigma-500, ZEISS). The tensile properties of various paper samples were tested by a DMA (TA-Q800) at strain rate of 100 μm·min^{-1}. The thermal properties of the samples were performed using thermogravimetric analysis (TGA) (TA Instruments Q500), and the paper samples were scanned from 35 to 750 °C with a heating rate of 10 °C/min under air atmosphere. The thermogravimetric analysis/infrared spectrometry (TG-IR) was performed using the TGA Q500 thermogravimetric analyzer which was coupled with the Nicolet 6700 FT-IR spectrophotometer via the transfer line, the heating rate is 20 °C min^{-1}. Electrical resistance transition behaviors of the various paper samples were recorded via connecting a multimeter (ESCORT 3146A) used typical two electrode method. Fire warning performance of paper samples (6 mm width and 20 mm length) was based on homemade alarm system via connecting with wires, an alarm lamp and a low voltage power supply (~24 V). The surface hydrophobic properties of various PU samples before and after hydrophobic treatment were obtained with a DSA30 CA analyzer (Kruss, Germany) using a 3 μL water droplet. The reciprocating friction and wear tests were conducted by a UMT-2 Universal Micro-Triboteste under room temperature condition. A friction velocity of 50 mm s^{-1}, friction distance of 10 mm and a normal

load of 0.5 N were applied to the specimen. The LOI values f PU composites coated FR coatings were measured using a JF-3 type oxygen index meter in accordance with ASTM Standard D2863-2009 (size: 100×10 ×10 mm^3). The combustion behaviors of the samples (100×100 ×10 mm^3) were carried out by a cone calorimeter device (Fire Testing Technology, UK) according to ISO Method 5660 under a heat flux of 35 kW/m^2. The infrared images of the PU foam samples during burning tests were recorded using an infrared camera (Fuluke Ti450 pro).

S2 Supplementary Figures and Tables

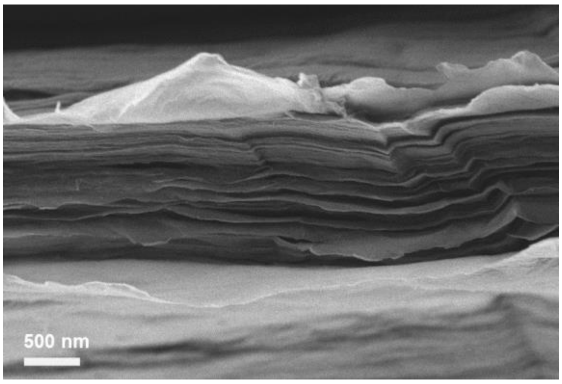

Fig. S1 Typical cross-sectional SEM image of pure GO paper, showing multi-layered structure

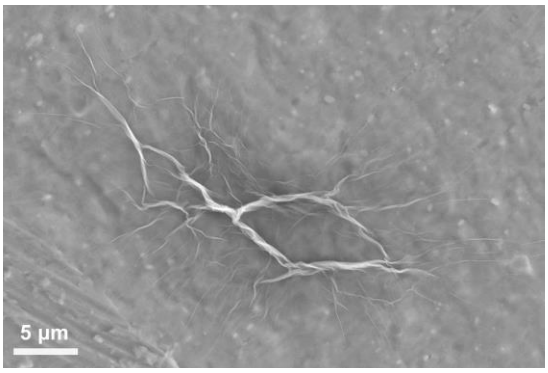

Fig. S2 Typical SEM image of GO sheet, showing wrinkled morphology structure

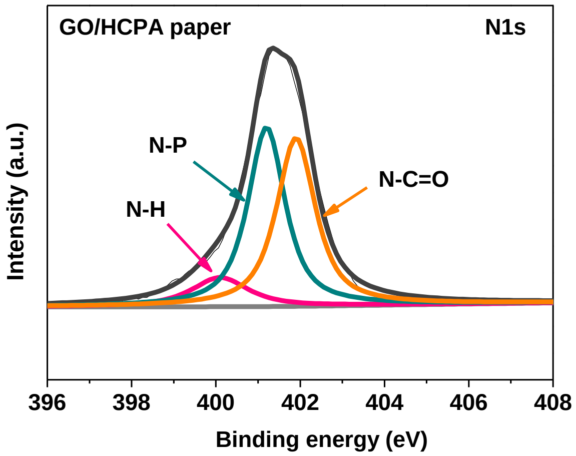

Fig. S3 XPS N1s spectra of GO/HCPA paper, indicating the existence of chemical interaction between GO and HCPA molecules

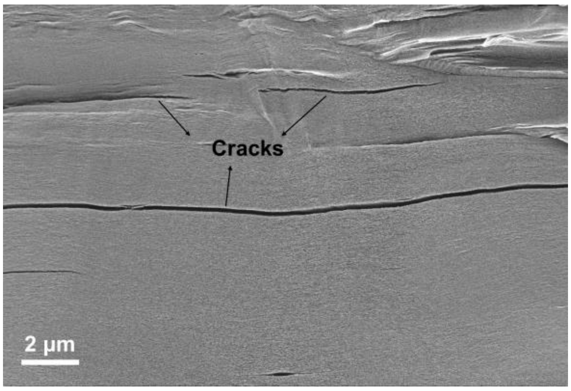

Fig. S4 Cross-sectional SEM image of G_1H_1 paper. Compared to $G_1H_{0.50}$ paper with compact layered structure, with a higher content of HCPA, the obvious cracks can be observed in cross-sectional structure of G_1H_1 paper, which may lead to the decreased mechanical strength, indicating that the bonding interaction among GO sheets becomes weak

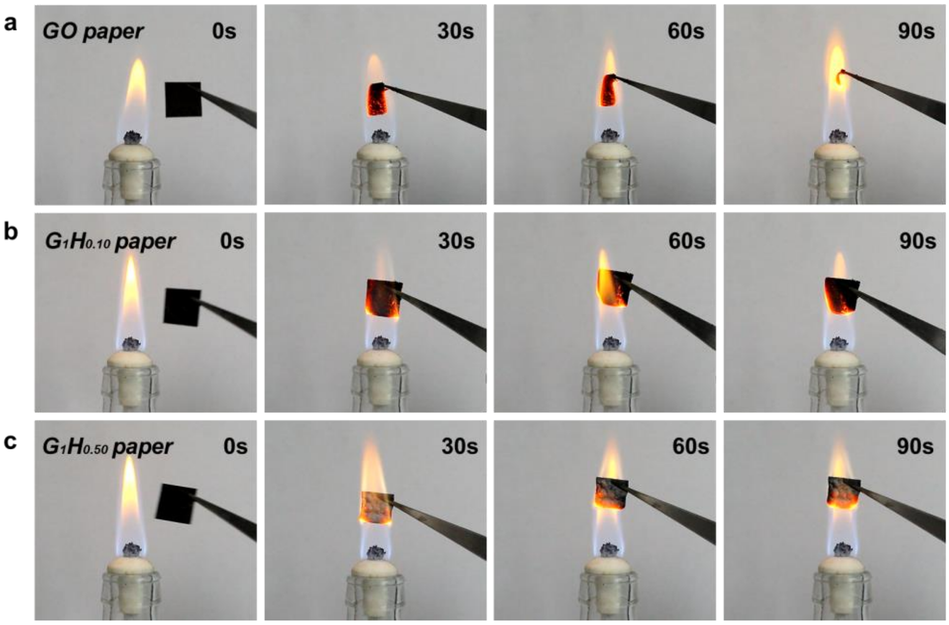

Fig. S5 Combustion process of various paper samples, showing improved flame retardancy with the addition of HCPA

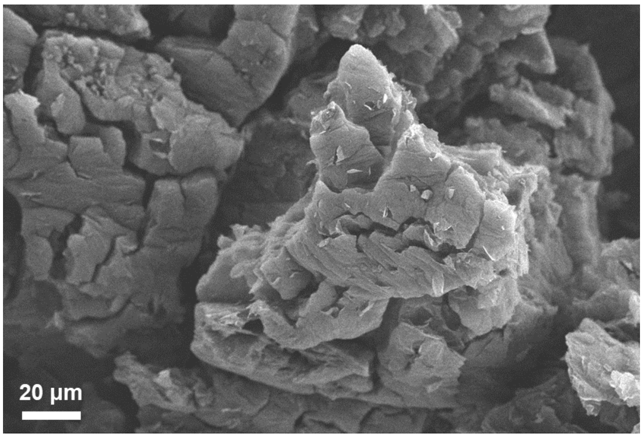

Fig. S6 Surface SEM image of pure GO paper after being burned, damaged structure can be easily observed and many obvious microcracks appeared on its surface zone, indicating its poor flame retardancy and thermal stability

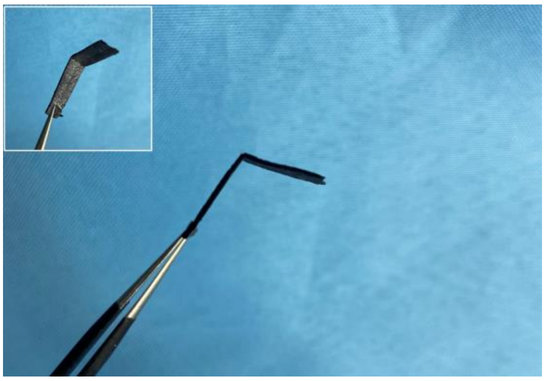

Fig. S7 Digital image of GO/HCPA paper after being burned, it still can be bent beyond 90°, showing a certain extent of structural stability

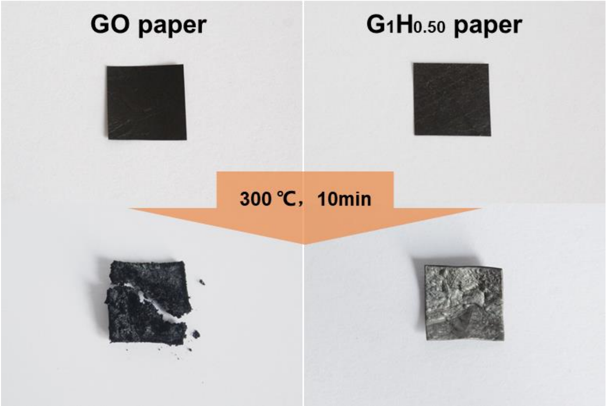

Fig. S8 Digital photographs of GO paper and GO/HCPA paper before and after 300 °C for 10 min. Compared with the damaged structure of pure GO paper after treatment, the structural integrity of GO/HCPA can well kept, indicating that the introduction of HCPA can improve thermal stability of GO network effectively

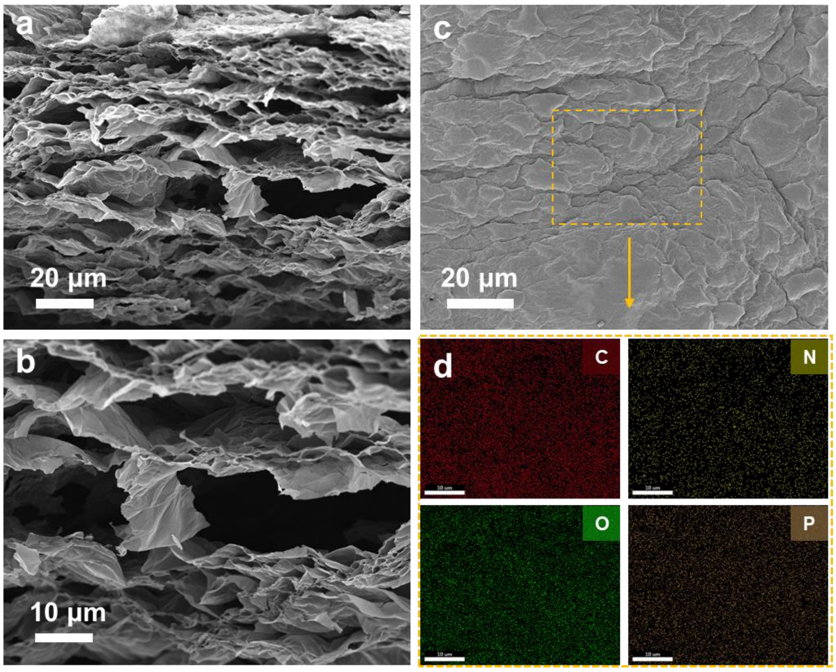

Fig. S9 **a**, **b** Typical cross-section and **c**, **d** surface SEM and corresponding EDS mapping images of $G_1H_{0.50}$ paper after burning, indicating P and N-doped phenomena in rGO network

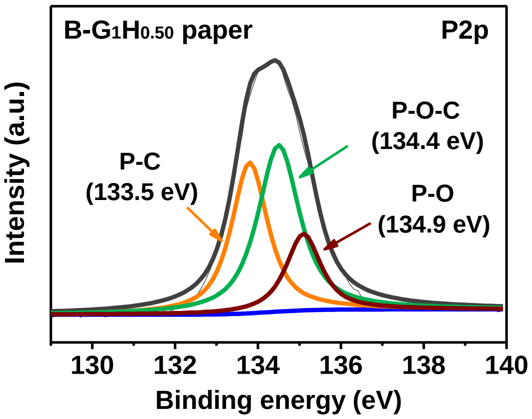

Fig. S10 XPS P2p spectra of $G_1H_{0.50}$ paper after being burned

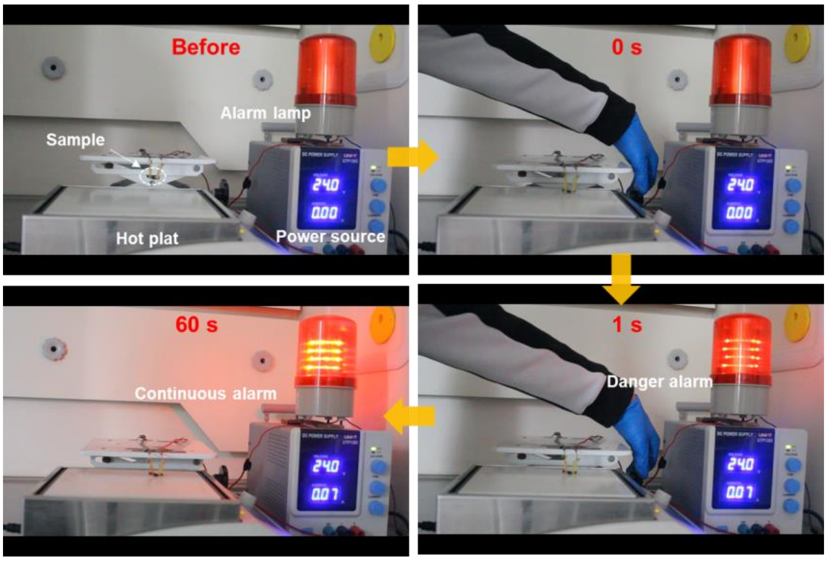

Fig. S11 Photographs of high-temperature warning process of $G_1H_{0.50}$ paper under the temperature of 350 °C, showing ultra-fast alarm response time of ~1s

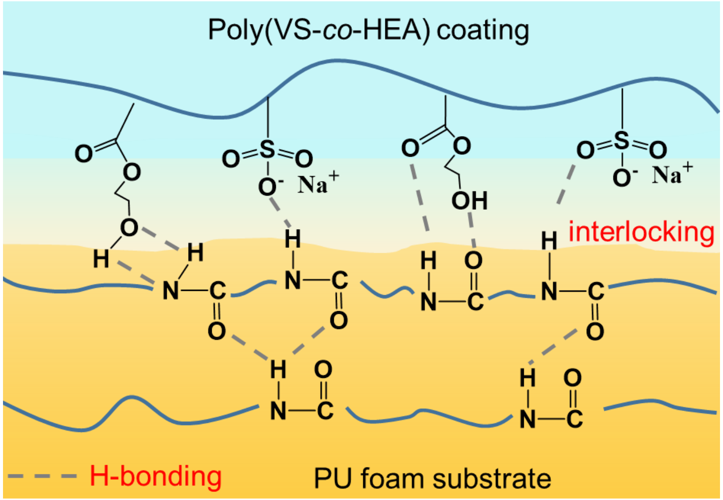

Fig. S12 Schematic illustration for interfacial H-bonding between poly(VS-co-HEA) and the PU foam substrate

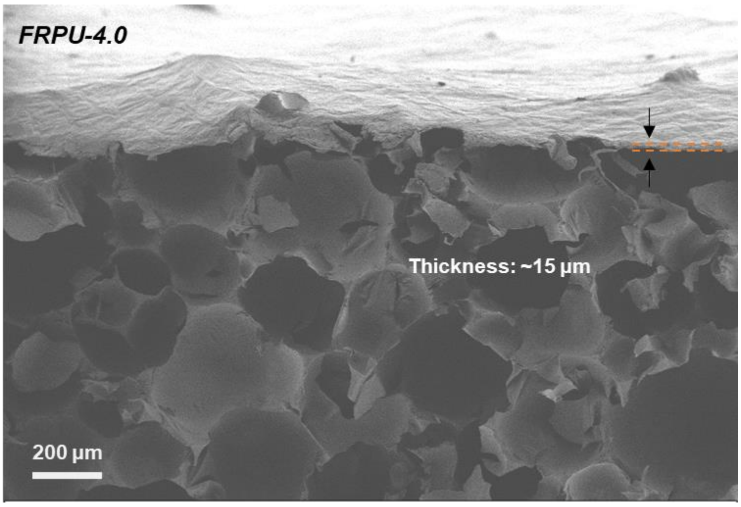

Fig. S13 Cross-section SEM image of PU foam coated with FR coatings

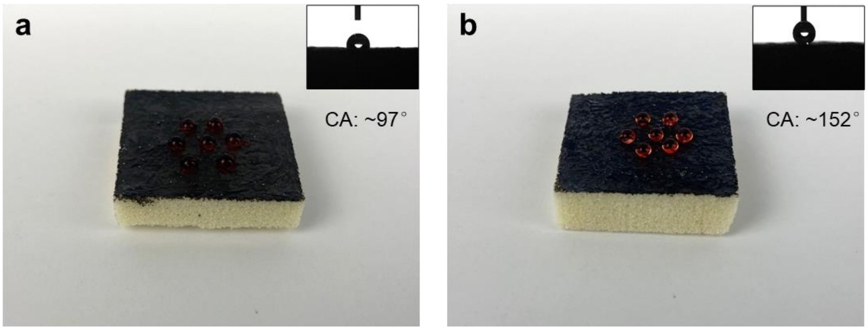

Fig. S14 Digital and corresponding contact angles photos (inset) of FRPU samples **a** before and **b** after hydrophobic treatment

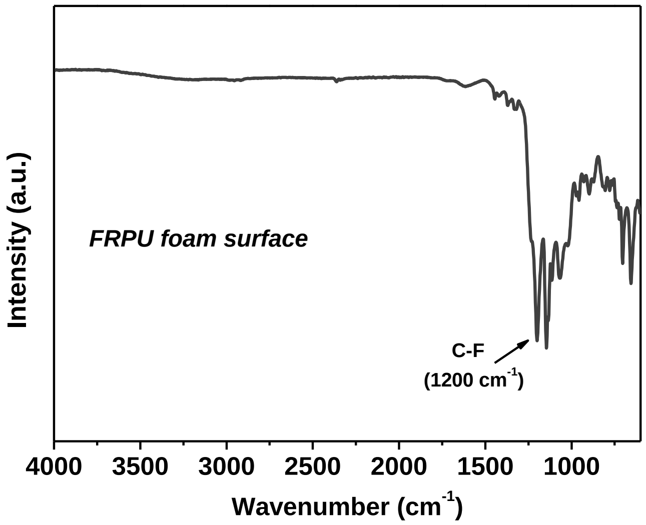

Fig. S15 IR spectra of hybrid flame-retardant coating on FRPU foam surface. Due to surface hydrophobic treatment, a strong peak at ~1200 cm^{-1} can be observed, which is assigned to C-F

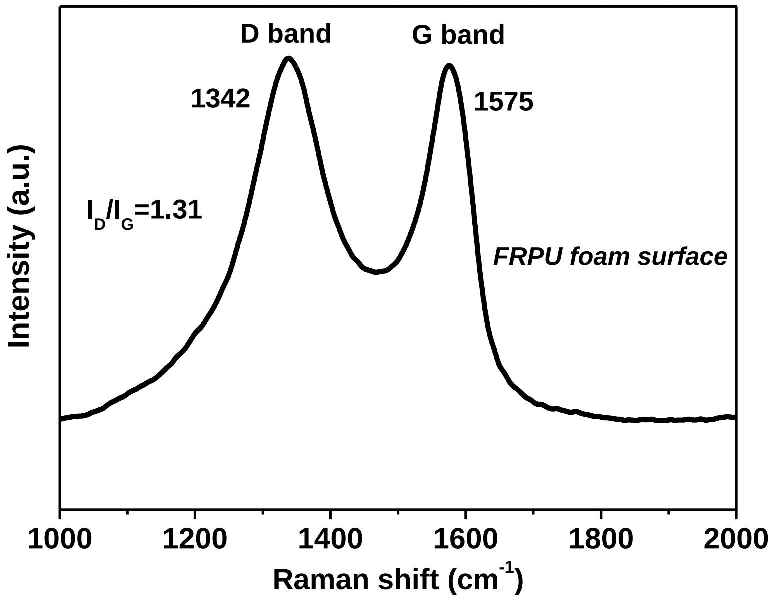

Fig. S16 Raman spectra of hybrid flame-retardant coating on FRPU foam surface. Compared to the GO/HCPA system, the characteristic D peak and G peak of hybrid coating system were shifted to lower wavenumber (from 1359 to 1342 cm^{-1} and 1604 to 1575 cm^{-1}, respectively), which may induce by surface hydrophobic treatment

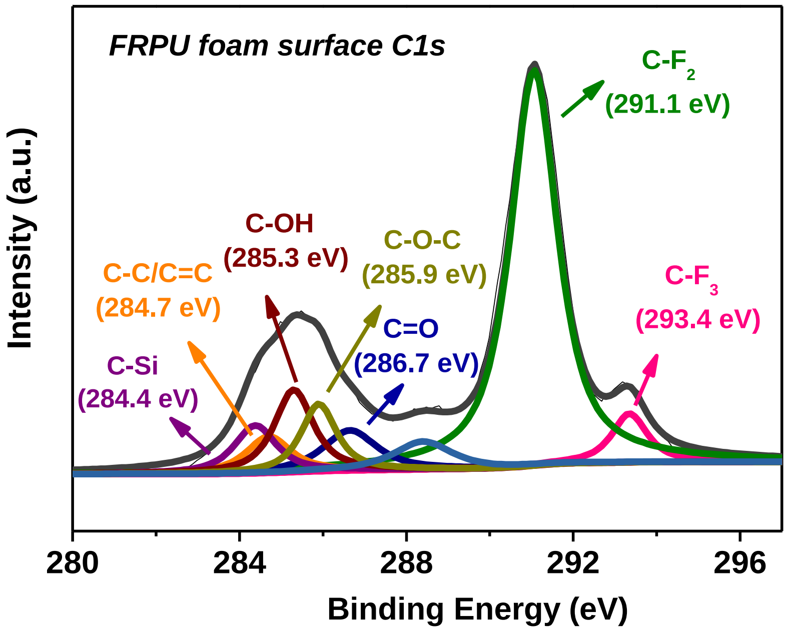

Fig. S17 XPS C1s spectra of hybrid flame-retardant coating on FRPU foam surface. The C1s core level spectrum shows shifted characteristic peaks of oxygen-containing groups and C-C/C=C, i.e., C-OH (284.7 eV), C-O-C (285.9 eV), C=O (286.7 eV), besides, the presence of C-Si, C-F_2, and C-F_3 bonds is attributed to saline chain, indicating the successful surface hydrophobic functionalization

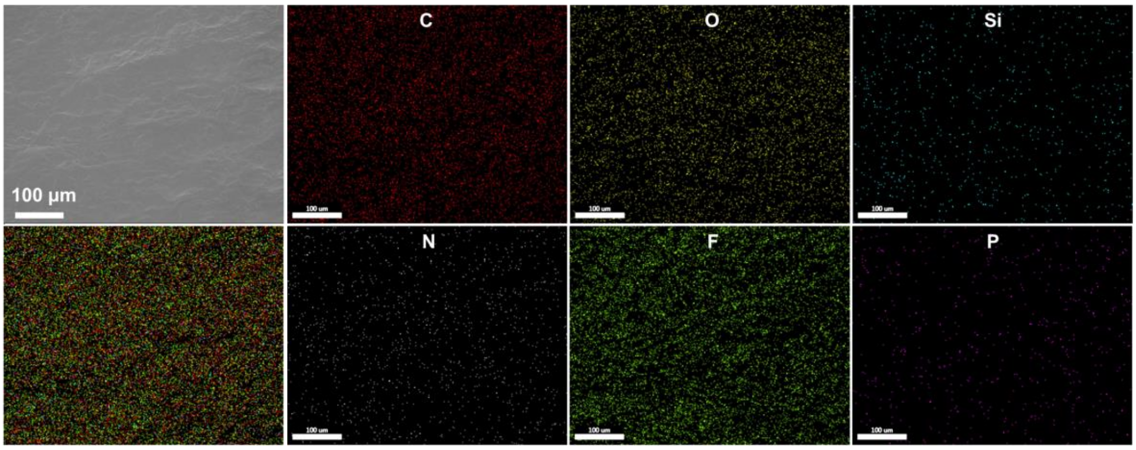

Fig. S18 Surface SEM image of hybrid flame-retardant coating on FRPU foam and corresponding EDS mapping images for C, N, O, F, Si and P, respectively. Evenly distribution of F and Si element can be observed on foam surface

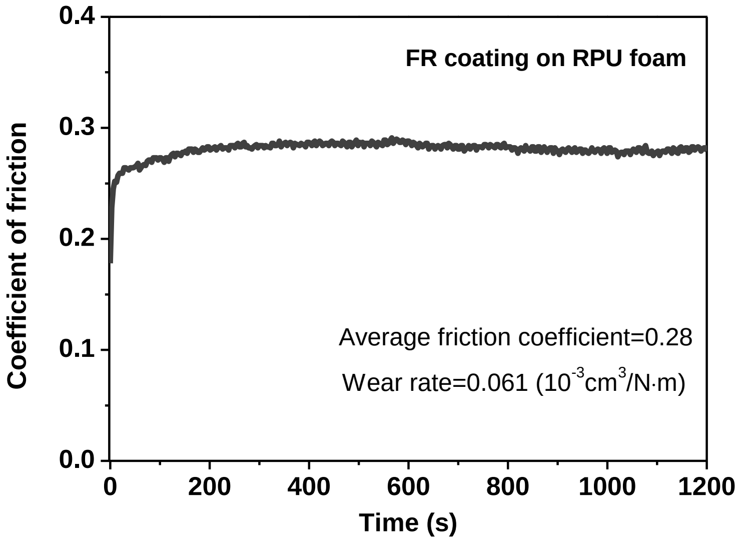

Fig. S19 Friction coefficient curve of FR coating coated on RPU foam surface. It can be found that the curve tends to be stable after 200 s, and the friction coefficient value remains almost no change in the later process. The result shows that the average friction coefficient value of FR coating is only about 0.28, besides, the wear rate is 0.061 (10^{-3} $cm^3/N{\cdot}m$), indicating a certain extent of wear resistance property of such FR coating

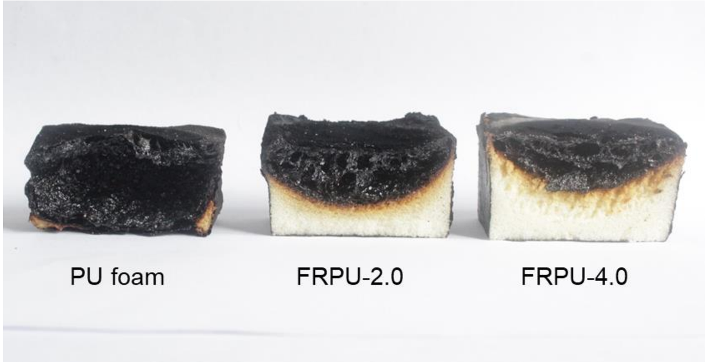

Fig. S20 Inner cross-sectional morphology of various PU foam samples after 30 min alcohol lamp flame attack

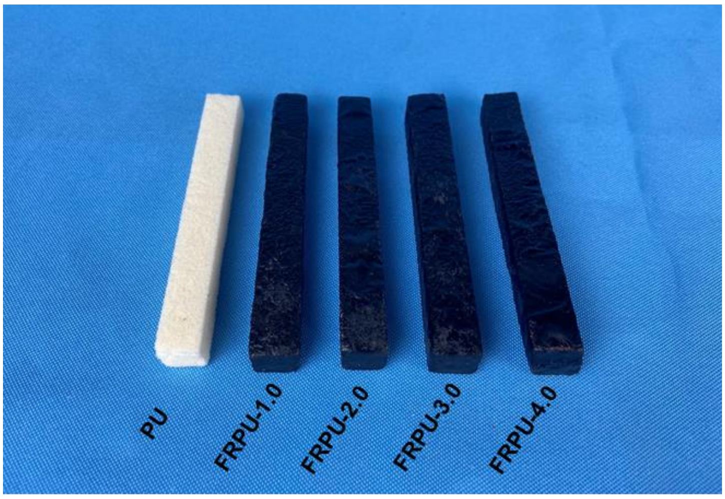

Fig. S21 Digital photos of various PU foam materials for LOI test

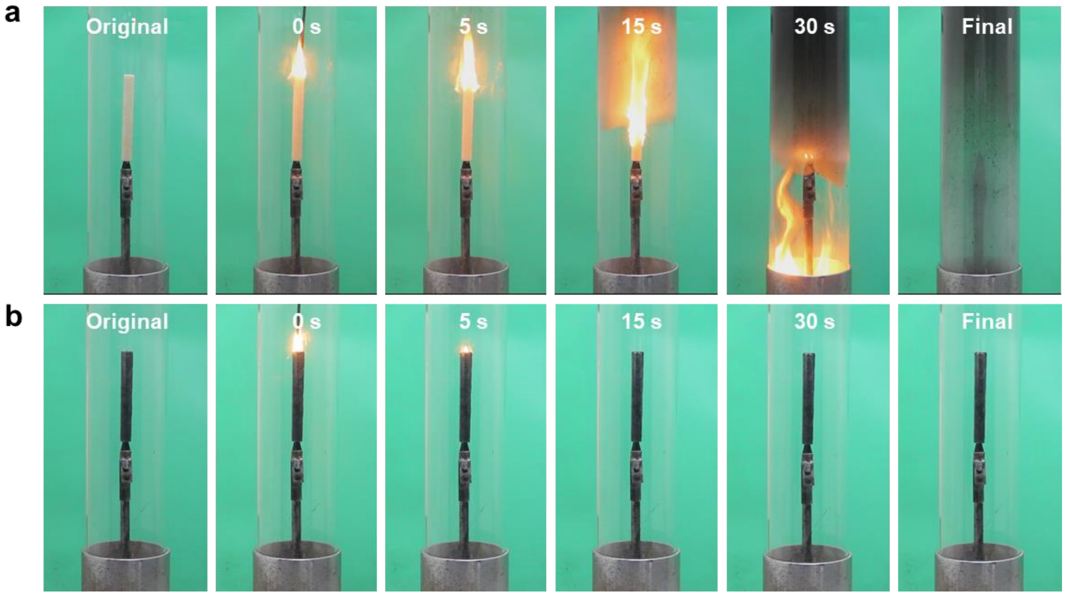

Fig. S22 Typical digital images of combustion behaviors of foam samples at a fixed oxygen concentration of 27% in the container: (**a**) pure PU foam and (**b**) FRPU-2.0 sample coated FR coating

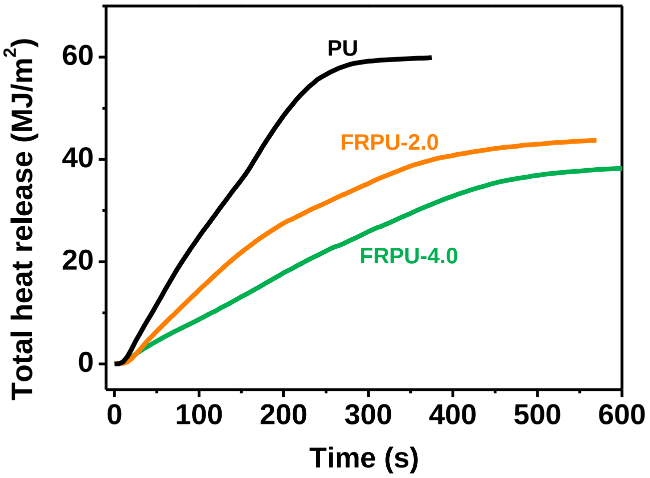

Fig. S23 Total heat release as a function of time of various PU foam materials

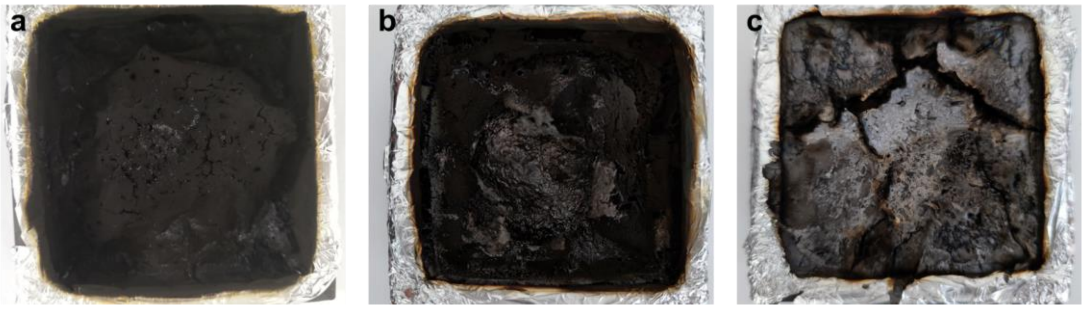

Fig. S24 Digital photos of residue chars for **a** pure RPU foam **b** FRPU-2.0 and **c** FRPU-4.0

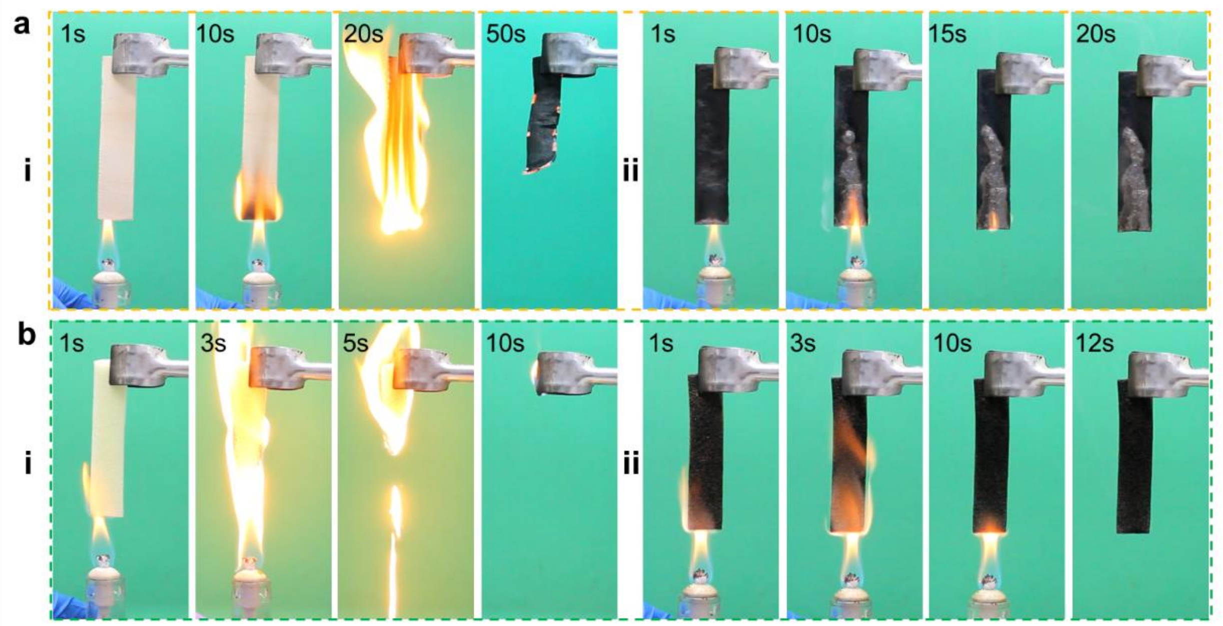

Fig. S25 Combustion behaviors of various samples. **a** (i) pristine natural wood and (ii) modified wood coated flame retardant coating with a content of 3 mg/cm^2; **b** (i) pure FPU foam (ii) and modified FPU foam coated flame retardant coating with a content of 20 wt%. Clearly, besides rigid PU foam material, such fireproof coating also exhibits desirable flame retardant efficiency when applied in other various combustible materials e.g., rigid natural substrate and flexible polymer foam substrate

Table S1 Tensile properties of pure GO paper and various GO/HCPA nanocomposite papers

Sample	Tensile strength (MPa)	Elongation at break (%)	Toughness (MJ/cm^3)
GO paper	40.9±3.6	1.2±0.2	2.46±0.2
$G_1H_{0.10}$ paper	53.6±5.6	1.3±0.2	3.94±0.4
$G_1H_{0.25}$ paper	68.8±7.4	1.6±0.3	6.0±0.7
$G_1H_{0.50}$ paper	94.7±8.3	2.2±0.2	13.9±1.8
G_1H_1 paper	58.5±3.3	1.7±0.2	5.6±0.4

Table S2 Comparison of mechanical strength of GO/HCPA paper and other similar GO-based paper nanocomposites

Sample	Tensile strength (MPa)	Elongation at break (%)	Toughness (MJ/cm^3)	Refs.
rGO/PDA paper	25.0	~2.6	NM	[S1]
Cellulose/GO film	~89	~5.9	NM	[S2]
Filtrated GO paper	~34	~0.13	NM	[S3]
PDDA-GO/ND paper	11.32	~0.38	4.71	[S4]
GO/TA/P-CNFs paper	~132	~1.2	NM	[S5]
ZHS/GO/PVC nanocomposite	14.3±0.3	NM	NM	[S6]
rGO paper (11)	~34	~3.0	NM	[S7]
SPI/MSF-g-COOH/CA/GN film	23.32	10	NM	[S8]
GO/BP-MoS_2 film	19.5	2.5	34.50	[S9]
GO/BP-NH_2 film	21.04	2.85	42.70	[S10]
GO/HCPA paper	94.7±8.3	2.2±0.2	13.9±1.8	***This work***

Notes: rGO: reduced graphene oxide; PDA: polydopamine; PDDA: poly (diallyldimethylammonium chloride); ND: nanodiamond; TA: tannic acid; P-CNFs: phosphorylated-cellulose nanofibrils; ZHS: zinc hydroxystannate; PVC: poly (vinyl chloride); rGO paper (11): reduced GO paper from GO suspension with PH value of 11; SPI: soy protein isolate; MSF-g-COOH: sisal cellulose microcrystals; CA: citric acid; GN: graphene nanosheets; BP: black phosphorene; MoS_2: molybdenum disulfide; BP-NH_2: amino-functionalized black phosphorene.

Table S3 Cone calorimetry data of PU, FRPU-2.0 and FRPU-4.0

Sample	pHRR (kW/m^2)	THR (MJ/m^2)	Residual mass (%)
PU	323	59.54	16.7
FRPU-2.0	203	43.83	42.0
FRPU-4.0	130	38.77	45.4

pHRR: peak heat release rate; **THR**: total heat release; **TSR**: total smoke release.

Table S4 The LOI value and UL94 rating of various foam samples

Sample	LOI (%)	UL94 rating
PU	18.4±0.3	NR
FRPU-1.0	26.7±0.4	NR
FRPU-2.0	30.4±0.3	V1
FRPU-3.0	33.7±0.4	V0
FRPU-4.0	36.5± 0.3	V0

Supplementary References

[S1] F. Luo, K. Wu, J. Shi, X. Du, X. Li et al., Green reduction of graphene oxide by polydopamine to a construct flexible film: superior flame retardancy and high thermal conductivity. J. Mater. Chem. A **5**(35), 18542-18550 (2017). https://doi.org/10.1039/c7ta04740a

[S2] A. Ahmed, B. Adak, T. Bansala, S. Mukhopadhyay, Green solvent processed cellulose/graphene oxide nanocomposite films with superior mechanical, thermal, and ultraviolet shielding properties. ACS Appl. Mater. Interfaces **12**(1), 1687-1697 (2020). https://doi.org/10.1021/acsami.9b19686

[S3] S. Liu, K. Hu, M. Cerruti, F. Barthelat, Ultra-stiff graphene oxide paper prepared by directed-flow vacuum filtration. Carbon **158**, 426-434 (2020). https://doi.org/10.1016/j.carbon.2019.11.007

[S4] B. Nan, K. Wu, Z. Qu, L. Xiao, C. Xu et al., A multifunctional thermal management paper based on functionalized graphene oxide nanosheets decorated with nanodiamond. Carbon **161**, 132-145 (2020). https://doi.org/10.1016/j.carbon.2020.01.056

[S5] C.F. Cao, B. Yu, B.F. Guo, W.J. Hu, F.N. Sun et al., Bio-inspired, sustainable and mechanically robust graphene oxide-based hybrid networks for efficient fire protection and warning. Chem. Eng. J. **439**, 134516 (2022). https://doi.org/10.1016/j.cej.2022.134516

[S6] T. Gao, L. Chen, Z. Li, L. Yu, Z. Wu et al., Preparation of zinc hydroxystannate-decorated graphene oxide nanohybrids and their synergistic reinforcement on reducing fire hazards of flexible poly (vinyl chloride). Nanoscale Res. Lett. **11**, 192 (2016). https://doi.org/10.1186/s11671-016-1403-z

[S7] P. Liu, H. Yang, X. Zhang, M. Jiang, Y. Duan et al., Controllable lateral contraction and mechanical performance of chemically reduced graphene oxide paper. Carbon **107**, 46-55 (2016). https://doi.org/10.1016/j.carbon.2016.05.050

[S8] Z. Zhang, D. Yang, H. Yang, Y. Li, S. Lu et al., A hydrophobic sisal cellulose microcrystal film for fire alarm sensors. Nano Lett. **21**(5), 2104-2110 (2021). https://doi.org/10.1021/acs.nanolett.0c04789

[S9] Z. Qu, C. Xu, X. Li, Y. Wu, K. Wang et al., Facile preparation of BP-MoS_2/GO composite films with excellent flame retardancy and ultrasensitive response for smart fire alarm. Chem. Eng. J. **426**, 130717 (2021). https://doi.org/10.1016/j.cej.2021.130717

[S10] Z. Qu, K. Wu, C Xu, Y. Li, E. Jiao et al., Facile construction of a flexible film with ultrahigh thermal conductivity and excellent flame retardancy for a smart fire alarm. Chem. Mater. **33**(9), 3228-3240 (2021). https://doi.org/10.1021/acs.chemmater.1c00113
